# User Experience in mHealth Research: Bibliometric Analysis of Trends and Developments (2007–2023)

**DOI:** 10.2196/75909

**Published:** 2025-11-10

**Authors:** Bashaer Alkhwaiter, Monira Aloud, Nora Almezeini

**Affiliations:** 1 Department of Management Information Systems, College of Business Administration, King Saud University, Building No. 3, Riyadh, 12371, Saudi Arabia, 966 118056577; 2 Department of E-Commerce, College of Administrative and Financial Sciences, Saudi Electronic University, Riyadh, Saudi Arabia

**Keywords:** bibliometric analysis, mHealth, origin lab, sitespace, systematic literature review, user experience, user satisfaction, VOS Viewer

## Abstract

**Background:**

The significance of mobile health (mHealth) apps transforms traditional health care delivery and enables individuals to actively manage their health. The success and effectiveness of mHealth apps heavily depend on the user experience and satisfaction. Previous studies have examined mHealth adoption through systematic literature reviews, focusing on mental health, chronic disease management, fitness, and public health responses to crises like the COVID-19 pandemic. However, the state of research, the key trends, themes, and gaps in the user experience and satisfaction with mHealth apps remain unexplored.

**Objective:**

This study aimed to investigate the state of research on user experience in mHealth apps through a bibliometric analysis. Furthermore, the study aims to systematically identify research trends and themes by extending the analysis of the science mapping technique, co-word analysis, and bibliographic coupling.

**Methods:**

The bibliographic data corpus was collected from Scopus and Web of Science and systematically analyzed using bibliometric performance analysis and science mapping techniques. The methodology incorporates various data processing and visualization tools, including VOS Viewer, OriginLab, and SiteSpace. Then, a comprehensive review metric, combining the PRISMA (Preferred Reporting Items for Systematic Reviews and Meta-Analyses) framework and a 4-step approach from data collection to interpretation is used.

**Results:**

The bibliographic analysis spans 16 years and includes 814 unique publications authored by 4870 researchers from 81 countries and 1948 organizations, published across 351 high-impact journals and prominent conferences. The analysis of research trends identifies 2 key trends: the differentiation in keyword usage for user experience and user satisfaction, and the research methodologies used within the domain. Furthermore, 5 research themes were identified exploring critical aspects of technology use, user engagement, and clinical integration. Although all 5 themes overlap, each theme focuses on distinct elements that help delineate their contributions to the overall understanding of mHealth apps: technological evaluation (Theme 1), design features for engagement (Theme 2), patient usability (Theme 3), long-term engagement factors (Theme 4), and clinical integration (Theme 5).

**Conclusions:**

This study offers a fundamental understanding of the bibliographic landscape of research on user experience and satisfaction with mHealth apps. By identifying major research clusters, influential works, and emerging topics, this analysis provides evidence-based guidance for researchers, developers, and health informatics practitioners. Furthermore, based on the research trends findings, future research should prioritize expanding the scope of user experience (UX) evaluation by incorporating diverse user populations, longitudinal studies, and emerging technologies such as artificial intelligence and personalized interventions. Integrating insights from interdisciplinary perspectives such as human-computer interaction, behavioral sciences, and health care informatics, the understanding of user needs and app effectiveness can be enhanced. A more standardized framework for assessing UX in mHealth apps is also recommended to facilitate comparability across studies and improve app design to maximize user engagement and health outcomes.

## Introduction

With the advancement of mobile technology and increased global connectivity, mobile health (mHealth) apps have become a significant tool in health care [1,2]. Modern access to medical information through mHealth apps allows users to monitor their biometrics and manage chronic illnesses effectively [3-5]. These mHealth apps are reshaping traditional health care delivery and empowering individuals to take an active role in managing their health [1,2,6,7]. In addressing health care access gaps, mHealth apps are vital, particularly in remote, underserved areas and during global crises such as the COVID-19 pandemic [8,9]. The importance of research in mHealth apps has only increased in recent years, as they present unique solutions for health and fitness, chronic disease management, and mental health support, making health care more accessible to diverse populations [10,11]. However, as mHealth apps become integrated into daily life, user experience and satisfaction have emerged as key factors in their effectiveness, with research revealing that engagement, usability, and personalization directly influence users’ sustained interaction with these apps [6,8,12-22].

Research in mHealth can be derived from 2 motivations: business opportunities and human impact. The digital health market is experiencing significant growth because of the increasing use of health apps for fitness tracking and chronic disease management. In 2023, global app downloads were projected to reach 257 billion, highlighting the pervasive role of mobile apps in everyday life [23]. Mobile app revenues are also expected to exceed US $613 billion by 2025, indicating substantial financial prospects [23]. Specifically, in the context of this research, health and fitness apps were projected to achieve 3.76 billion downloads in 2022, with medical apps reaching 305.4 million downloads [24,25]. These figures underscore the strong global demand for mobile health solutions. Concurrently, the human impact is equally influential, with health and wellness becoming priorities for individuals and governments alike. Initiatives are increasingly focused on promoting patient empowerment and improving health care accessibility, as underscored by World Health Organization’s 2022 framework, which emphasizes the importance of active patient engagement in health decisions [26].

Understanding how and why people adopt mHealth solutions is of critical significance. On an individual level, effective digital tools may promote healthier lifestyles, increase adherence to treatment plans, enhance patient education, and ultimately improve health outcomes [3-5]. On a societal level, the large-scale adoption of mHealth apps and wearables can ease the burden on health care systems, reduce costs, and improve health equity by delivering accessible and personalized care [1,2,6,7]. However, to realize these benefits, it is essential to investigate the factors that shape user experience and satisfaction with mHealth apps [6,8,12-22]. Research in this area aims to ensure effective engagement, address issues of interoperability and integration with clinical workflows, and develop strategies that sustain long-term usage and trust. However, to build on insights from technology development, health, behavioral science, and user experience design, research in this area faces a critical gap in fully mapping the intellectual structure, theoretical foundations, and interdisciplinary perspectives shaping this evolving body of knowledge.

Previous research has tried to bridge this gap by reviewing the literature influencing various mHealth apps and wearables. Using a systematic literature review method to examine mHealth adoption, user experience, and satisfaction, tailored to the contexts of mental health [15,19,27-39] and chronic disease management [6,40-49], fitness apps [8,50-52], and public health responses to crises like the COVID-19 pandemic [53-57]. Most reviews targeted mHealth for specific demographic groups, including women [58], older adults [59], and children [60,61]. Some reviews have adopted a broader scope, addressing general mHealth topics over extended periods [62-67]. However, the studies incorporated in these reviews often involved small sample sizes (2‐178 studies), which may limit the representativeness and generalizability of the conclusions. A larger number of reviewed studies (n=365) were included in the study by Hu et al [68], the only bibliometric analysis identified, while offering a distinct perspective on the impact of research in this area, health wearables were the main focus. Collectively, these studies underscore the importance of considering user satisfaction, engagement, the usability of mHealth, and the impact of content and design on user experience. This suggests that future digital health solutions must adapt to user needs and preferences to maximize their impact, requiring ongoing collaboration between researchers, designers, and end users to refine and optimize these mHealth innovations. These insights are crucial for overcoming current limitations and enhancing the efficacy of digital solutions in health care.

Despite this helpful effort in reviewing related literature, a clear intellectual structure has not yet been offered. To address this gap, a more comprehensive review is required to investigate the bibliographic contributions to user experience and satisfaction with mHealth solutions. We included both user experience and user satisfaction as keywords to capture their combined and distinct usage within the same body of literature. Therefore, this bibliographic analysis explores the state of research on user experience and satisfaction with mHealth apps (UXS-mHealth apps). Aiming to exhibit the intellectual structure of related research in this area, this study identifies the leading publications, authors, countries, organizations, and sources that contribute to understanding mHealth apps and impact research development in UXS-mHealth apps. Furthermore, this study explores the research trends within thematic research areas related to UXS-mHealth apps via a systematic qualitative review. By integrating these quantitative and qualitative review methods, this review offers a data-driven perspective that complements traditional literature reviews and enables a more holistic understanding of the research on UXS-mHealth apps. These findings will contribute to the academic discourse and provide practical insights for developers, designers, and policymakers who aim to improve the effectiveness and user-friendliness of mHealth apps.

## Methods

### Overview

This study adopts a bibliometric analysis in conjunction with a systematic literature review (SLR). The bibliometric analysis is undertaken to map the current state of research on UXS-mHealth apps and to identify the most influential publications and authors in the field. Building on these findings, the SLR offers an in-depth examination of literature, identifying the evolution of research themes, highlighting emerging topics, and revealing gaps that warrant further investigation.

### Bibliographic Analysis

**Figure 1.**
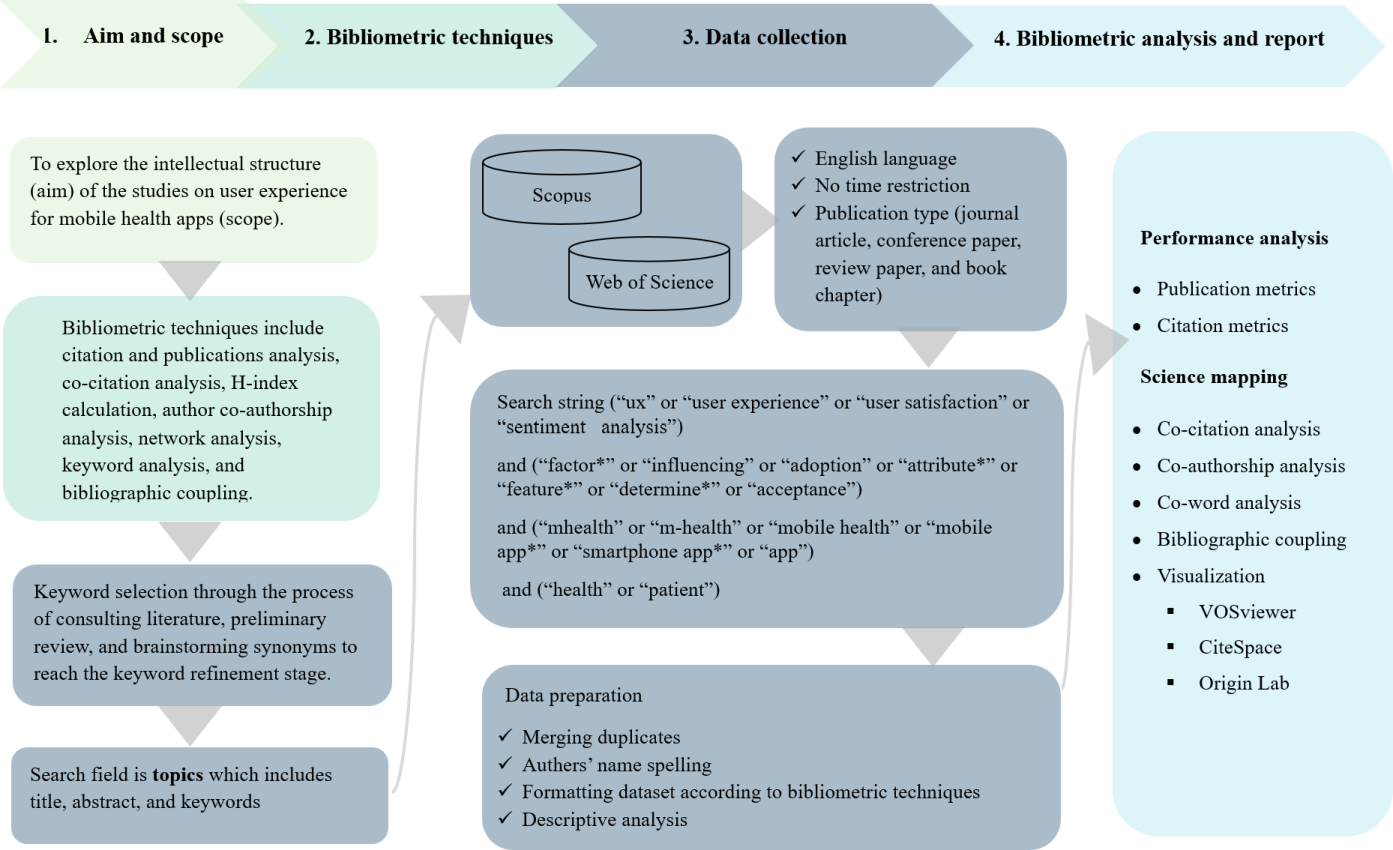
The 4-step bibliometric analysis search design used in studying the research structure of user experience and satisfaction with mobile health (mHealth) apps.

The bibliometric analysis follows the procedural guidance established in the study by Donthu et al [69]. Figure 1 illustrates the bibliometric analysis process, including defining study aims, selecting techniques, data collection, and conducting analysis. This framework ensures transparency and reproducibility when analyzing UXS-mHealth apps.

#### Data Sources and Search Strategy

The data sources used in this analysis are Scopus and Web of Science. The focus on retrieving bibliographic data for publications indexed in the established databases, Scopus and Web of Science, ensures the inclusion of high-quality and widely recognized sources to explore the field development. Scopus and Web of Science are widely considered the premier databases for conducting bibliometric research because of their comprehensive coverage, rigorous indexing criteria, and multidisciplinary scope [69]. Both platforms offer robust citation tracking capabilities, essential for a thorough bibliometric assessment, enabling the identification of influential papers and the evaluation of research impact [70]. Furthermore, rigorous selection criteria and quality control mechanisms ensure the inclusion of reputable journals and high-quality research [71]. Including both databases eliminates bias and provides access to various scholarly articles, conference papers, and book chapters, ensuring researchers can capture a broad and representative sample of relevant literature [72]. In addition, Web of Science and Scopus provide advanced analytical tools for examining citation networks, author metrics, and journal impact, which are critical for detailed bibliometric studies [73]. Other databases may not offer the same level of comprehensive coverage or advanced analytical features, potentially limiting the scope and depth of bibliometric analyses [10]. Thus, selecting these 2 databases addresses the study’s aim because their inclusion of citation data allows for sophisticated analyses of research impact, trends, and collaborations, making them indispensable resources for bibliometric studies [69,71].

From both platforms, the bibliographic data were extracted for each publication. This included authorship, publication year, journal name, affiliation, document type, volume, issue, pages, citations, and sponsorship. This data was gathered using the export functions embedded in Scopus and Web of Science with full record selection, complemented by manual checks to verify accuracy.

#### Inclusion and Exclusion Criteria

The inclusion and exclusion criteria in this study ensure that the research captures a comprehensive and representative literature sample designed based on the study’s objectives. These criteria involve several dimensions: publication type, language, data source, keywords, and search field. The publication type inclusion criteria include journal articles, conference papers, review papers, and book chapters. Only publications indexed in Scopus and Web of Science were considered, and all search results were limited to publications published in English, without restriction on publication date. The search was done in June 2024. The selection is restricted to full-text articles to facilitate comprehensive analysis and ensure complete bibliographic and citation data availability.

The selection of relevant keywords began with a comprehensive review of the literature, focusing on titles, keywords, and abstracts of highly cited and pertinent articles. After analyzing over 20 articles, including [3,4,10,15,18,74,75], it became evident that many commonly used keywords were already present in the initial search string. To enhance the search’s alignment with the study’s focus, additional keywords were brainstormed by identifying core concepts, such as user experience, user satisfaction, and mHealth apps, followed by expanding these concepts with synonyms and related terms. This iterative process ensured a comprehensive and targeted search string that effectively captured the essential aspects of the research topic.

The search string is categorized into 4 groups: A, B, C, and D. Group A includes user experience and user satisfaction, whereas those concerning the identification of influences, factors, and determinants are included in Group B. Groups C and D focus on keywords specific to the mHealth domain and health and patient contexts, respectively. The advanced search tools in Scopus and Web of Science use the Boolean operator AND to connect the groups, and OR was used within each group to enhance the specificity and relevance of the search results. These criteria ensure that the bibliometric analysis is focused, relevant, and based on high-quality sources. After a preliminary review of the relevance of the abstract, some keywords were excluded. The exclusion of keywords from each group is illustrated in the following:

- Group A: “UX” OR “user experience” OR “user satisfaction” OR “sentiment analysis.” The keyword “Ranking” was excluded as it predominantly related to ranking methodologies rather than user experience or satisfaction.
- Group B: “factor*” OR “influencing” OR “adoption” OR “attribute*” OR “feature*” OR “determine*” OR “acceptance.” The keywords “use,” “usage,” and “perceived value” were excluded because they predominantly led to publications on the clinical use of mHealth systems and perceived value in clinical contexts, which were outside the review’s scope.
- Group C: “mhealth” OR “m-health” OR “mobile health” OR “mobile app*” OR “smartphone app*” OR “app.” The keywords “app*” and “system*” or “digital platform*” were excluded as they primarily retrieved publications on electronic clinical systems or web-based platforms, which were not relevant to mHealth apps.
- Group D: “health” OR “patient.”

#### Data Preparation

Data preparation is crucial for aligning the data corpus with the bibliometric techniques chosen, as recommended by [69]. The data cleansing process includes merging duplicate publications within and between databases, checking for variations in authors’ name spellings, and processing author affiliations. A manual review is conducted to reconcile discrepancies such as differences in initials, name order, or the inclusion of middle names. In addition, author identifiers are used to ensure the precise identification and merging of publications by the same author, regardless of variations and the indexed platform. Furthermore, the preparation process involves verifying the publication type, where publications initially categorized as review papers, such as those reviewing apps, are reclassified as journal or conference papers. This reclassification ensures that publications are accurately categorized according to content and format. The tools used for data preparation include Microsoft Excel for manual checks, and CiteSpace (6.4.R1-64-bit-Advanced) and Zotero for merging duplicates. This approach of preparing data will effectively address the study’s aim of exploring the state of research on user experience in mHealth apps by enabling the filtration of journals, publication type trends, time trends, subject areas, author productivity, and citation metrics.

### Systematic Literature Review

The bibliometrics is extended for further exploration of co-word analysis and bibliographic coupling by using the PRISMA (Preferred Reporting Items for Systematic Reviews and Meta-Analyses) framework [76] with a 4-step approach from data collection to interpretation [77].

**Figure 2.**
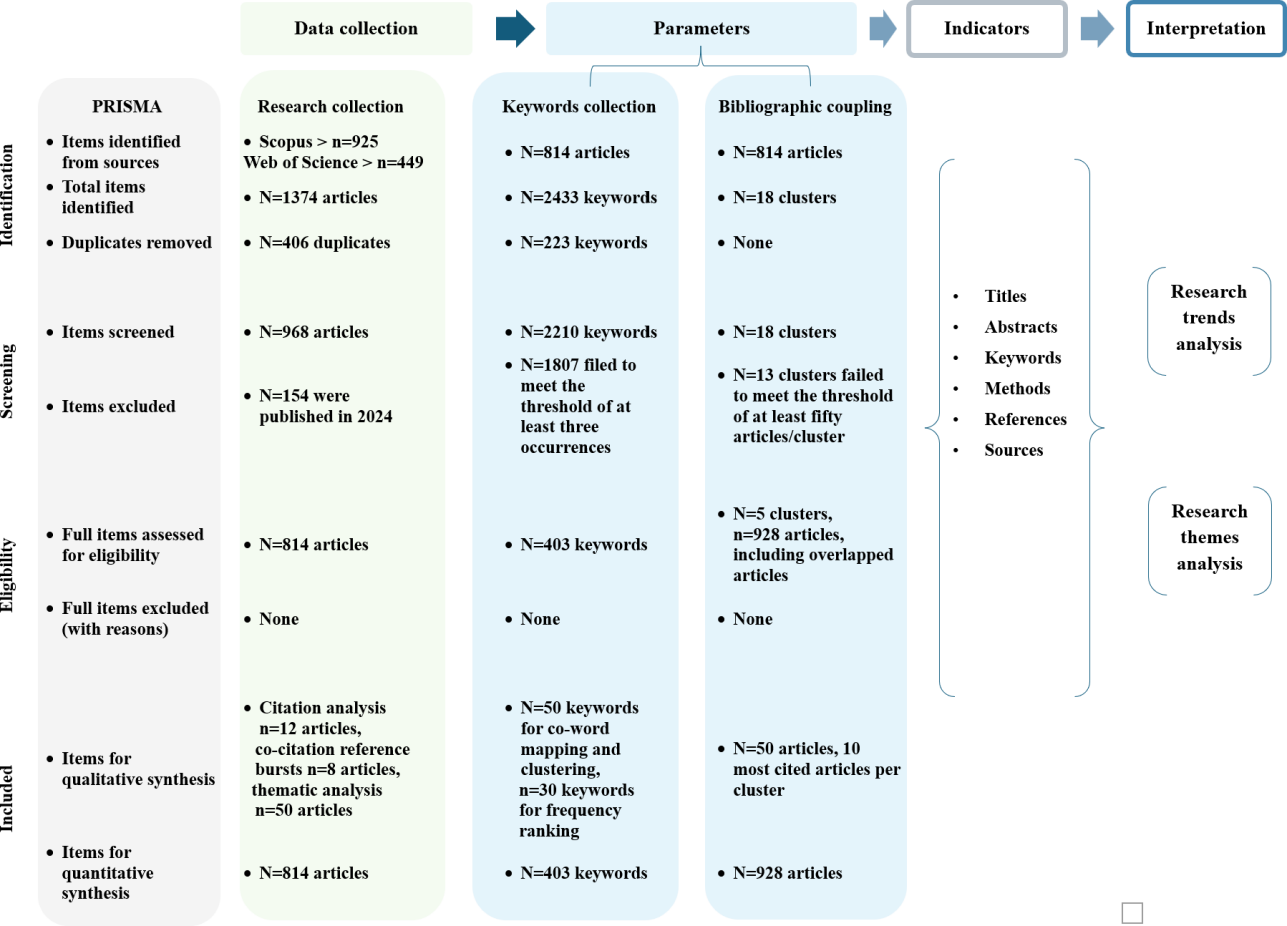
Methodological workflow for mHealth literature synthesis (2007‐2023) using PRISMA (Preferred Reporting Items for Systematic Reviews and Meta-Analyses)-guided selection and the data collection to interpretation process.

In Figure 2, the PRISMA flow diagram is represented vertically and outlines the vertical systematic filtration process involving identification, screening, eligibility, and inclusion phases. This framework is applied to the horizontal process of data collection, which involves defining parameters and indicators and the interpretation of results. This process is used to identify meaningful conceptual linkages among the collected data. For each publication, indicators such as titles, abstracts, keywords, research methods, research type, and sources are systematically extracted to characterize and categorize the literature. The PRISMA checklist can be found in Checklist 1.

## Results

The bibliographic dataset encompasses 1376 search results from Scopus and Web of Science, including unique and overlapping results across the two databases. Of the 1376 results, 42% (407/1376) are duplicates, while Web of Science contributes 4.4% (43/1376) of unique results, and Scopus contributes (53.6%, 519/1376). The distribution of sources and different document types is illustrated in Multimedia Appendix 1.

### Bibliographic Performance Analysis

#### Annual Trend Analysis

To investigate the intellectual framework of research on UXS-mHealth apps, the annual trend analysis provides valuable insights into the progression of research activity over time. The 2 most commonly used metrics are the annual total number of publications (TP) and total citations (TC); publications reflect research productivity, whereas citations measure research impact [69]. By examining these metrics, it becomes evident how the field has grown, contracted, or responded to influential events over time [69,78].

**Figure 3.**
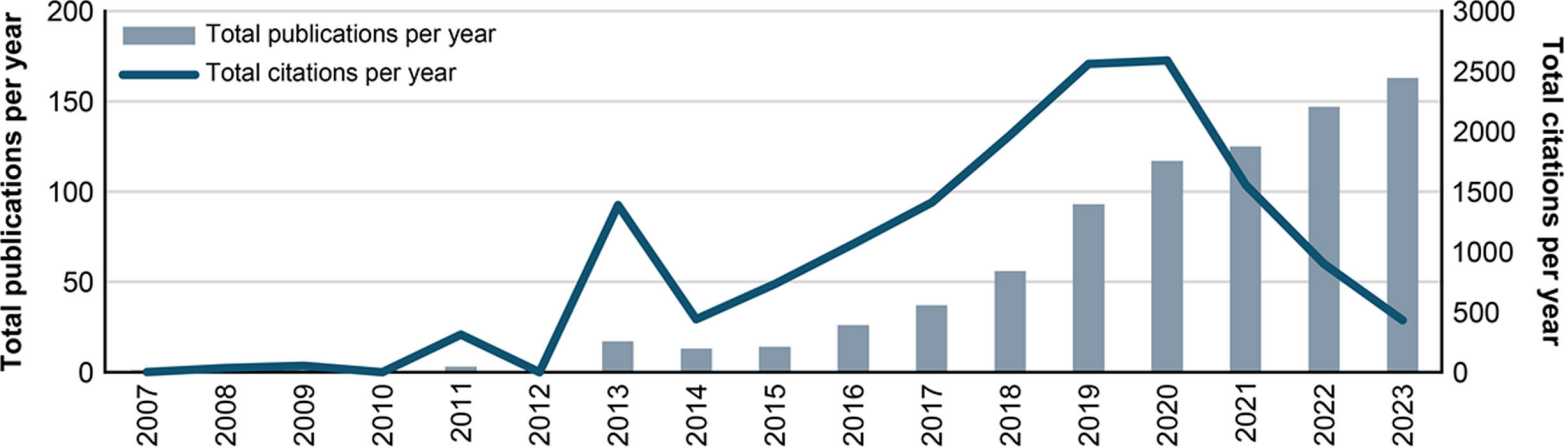
The annual analysis of publications on UXS-mHealth apps from 2007 to 2023 shows the productivity of publications and the research impact based on the annual total publications (TP) and total citations (TC).

This study’s annual analysis of the 814 publications from 2007 to 2023 reveals distinct patterns that illustrate the growth of the UXS-mHealth apps domain, as shown in Figure 3. The dataset’s first publication year is 2007, although it covers an unrestricted time span. This event may indicate a connection to technological advancement in 2007, synchronized with Apple’s launch of the first interactive smartphone. The launch of the iPhone marked a significant shift in mobile technology, making smartphones more accessible and elevating the importance of mobile apps [79,80]. This technological milestone is evident in the initiation of academic output, as scholars began exploring the broader implications of smartphone technology across various fields.

From 2007 to 2012, publications primarily focused on mobile user interactions and apps. In 2013, the field experienced a significant peak, signaling a surge of interest or noteworthy advancements that captured researchers’ attention. This might have been due to the rising competition between iOS and Android operating systems, as Google released the Android Play Store around this time [79,80]. However, this was followed by a decline in 2014, which may suggest a temporary shift in focus, challenges in the field, or the resolution of particular research questions [78]. During this decline, most publications concentrated on fundamental concepts of mobile app acceptance and usability. However, an innovation breakthrough, such as the announcement of the Apple Watch in 2014, designed to track health and fitness metrics, likely shifted research interest [81].

From 2015 onward, a steady increase in publications indicates sustained and growing interest in UXS-mHealth apps. The number of research efforts based on annual TP increased from 17 in 2013 to 163 in 2023. This consistent rise in TP from 2015 to 2023 could be attributed to technological advancements, methodology, or emerging mobile apps that have evolved research efforts [69]. These trends indicate that the field is progressing and expanding its impact as it matures, attracting contributions from a growing number of researchers.

The citation trend depicted in Figure 3 shows an annual increase in citations, except for two declines in 2014 and between 2021 and 2023. There is also a slight increase in citations during COVID-19, followed by a decrease. The heightened attention during this period led to a rapid increase in research dissemination, which resulted in many papers being cited quickly [82]. The decline in citations for recent publications between 2021 and 2023 is often due to the time required for new publications to gain visibility within the academic community [83-85]. The citation count for new papers is still evolving and cannot be fairly compared to older publications that have had more time to accumulate citations [83-85].

Overall, the gradual evolution of the field underscores the developing stages of research into UXS-mHealth apps. This analysis provides valuable insights into the development of this area, revealing both promising opportunities for future research and aspects that warrant further exploration. As the field advances, understanding these stages can guide researchers in addressing emerging challenges and leveraging new technologies, ultimately enhancing user experience on mobile app outcomes.

#### Leading Countries Contributions

The analysis of regional distribution and collaborative networks for UXS-mHealth apps publications from 2007 to 2023 reveals several key trends that enhance our understanding of geographical productivity and impact. Countries contributing to the research were collected from the authors’ affiliations. Table 1 presents the 20 top contributing countries based on the TP, TC, citations per publication (TC/TP), and the measure of influence h-index (h). Additional visualization for countries, ranked based on the TP and citations, is presented in Multimedia Appendix 2. The distribution of these outputs highlights how different countries have shaped research within this domain.

The United States leads in most metrics, collaborating primarily with Ireland, Switzerland, the United Kingdom, Australia, and others. In terms of impact, the United States (h=30) is the most influential, followed by the United Kingdom and Australia (h=20). The United States accounts for 30.47% (248/814) of the TP, followed closely by the United Kingdom, Australia, and China. However, a different pattern emerges when analyzing the citations per publication (TC/TP): Saudi Arabia, Portugal, and the United Kingdom surpass other nations, coming to the forefront. Notably, Saudi Arabia and Portugal stand out due to citations in publications despite having fewer publications. Saudi Arabia, in particular, demonstrates a unique position with relatively few publications but a strong influence on the field. This impact is further emphasized by Saudi Arabia’s collaborative strength, which ranks fifth internationally. This suggests that strategic international collaborations can enhance a country’s influence and citation impact, even with lower research output.

**Figure 4.**
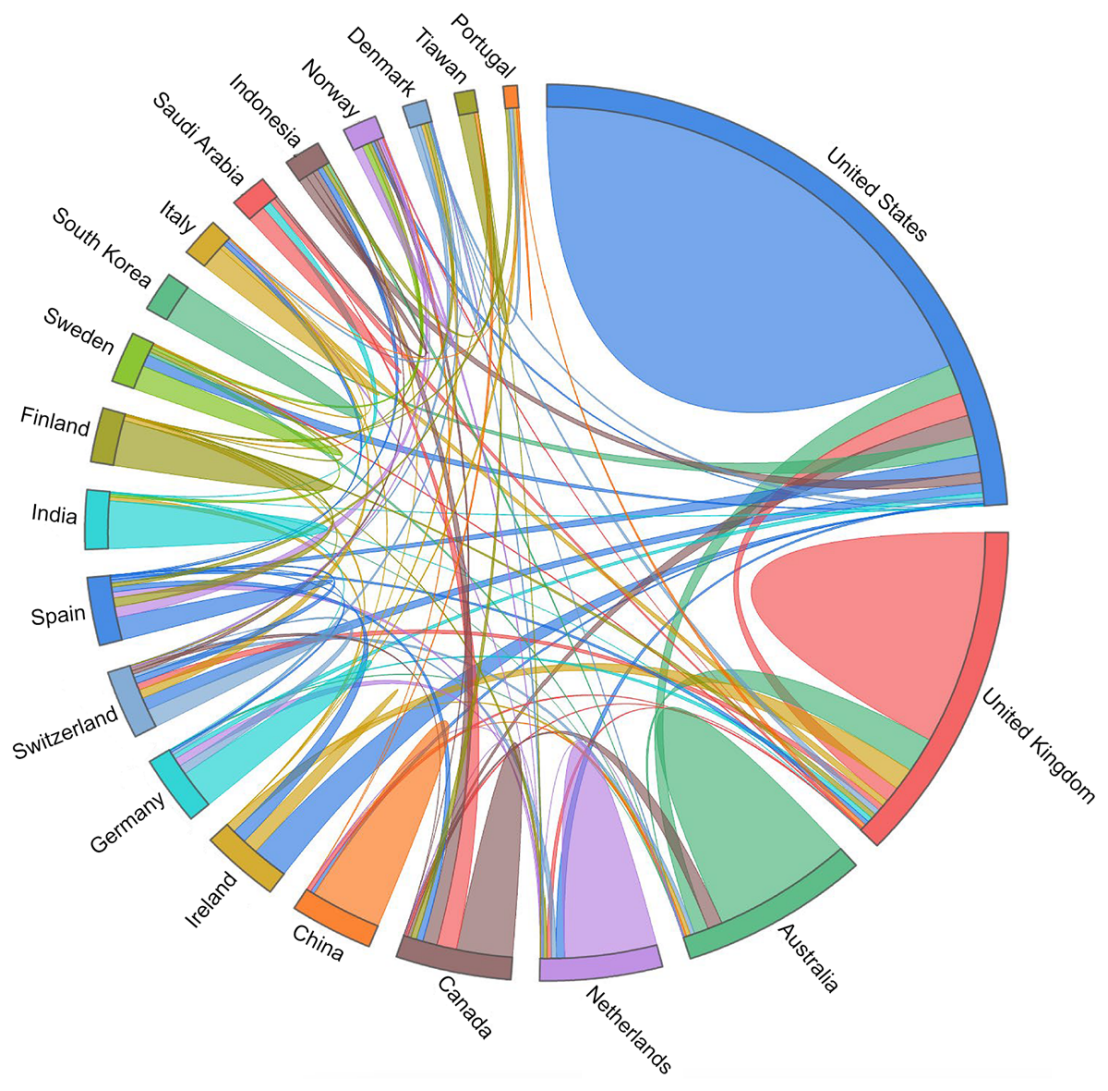
A chord diagram illustrating the top 20 countries’ publication contributions and collaboration networks in user experience and satisfaction with mobile health apps, with the thickness of the connecting chords representing the number of citations.

The chord diagram in Figure 4 visualizes the collaboration between the top 20 countries contributing to the UXS-mHealth apps research. The diagram was produced using the OriginLab software package for visualization purposes. The size of each segment corresponds to the total number of research publications produced by each country. The more significant segments indicate higher output, indicating the most prolific countries in the research area. The collaborative links between countries are depicted as lines or chords, connecting the segments. These lines symbolize coauthorships or joint research projects between nations. Thicker lines reflect more highly cited collaborations, indicating that the collaborative efforts between certain countries significantly influence the research landscape.

**Table 1.** Contribution of the top 20 countries, out of 81, to research on UXS-mHealth apps from 2007 to 2023. The country ranking is based on two bibliometric indicators: total publications (TP), total citations (TC), and the measure of influence h-Index.

Country	TP	TC	(TC/TP)	h-Index
United States	248	4275	17.24	32
United Kingdom	103	1971	19.14	20
Australia	87	1357	15.60	20
China	75	558	7.44	13
Germany	64	809	12.64	14
Netherlands	60	593	9.88	14
Canada	56	542	9.68	13
Spain	46	405	8.80	13
India	45	262	5.82	9
Italy	37	428	11.57	8
Switzerland	34	137	4.03	8
Indonesia	29	226	7.79	5
South Korea	27	120	4.44	8
Denmark	24	115	4.79	4
Ireland	23	314	13.65	8
Portugal	21	487	23.19	4
Sweden	21	223	10.62	8
Saudi Arabia	20	464	23.20	7
Norway	19	310	16.32	6
Taiwan	19	237	12.47	6

Furthermore, the chord diagram represents the proportion of international collaborations relative to local, independent work for each country. This feature visualizes the proportion of research involving international cooperation relative to domestic efforts. Therefore, collaboration patterns, in countries such as China, India, Finland, South Korea, and Taiwan, exhibit a more localized approach to collaboration. While these nations are productive in their research output, their collaborative networks remain primarily confined to their local regions. These countries’ focus on local connections might restrict their influence in a broader global context. The country cooperation network shown in Figure 4 is obtained using the Origin tool through affiliation analysis. This visualization offers more profound insights into a country’s research volume and impact and the balance between local and global collaboration.

These findings underscore the importance of productivity and collaboration in shaping regional contributions and global influence within a specific research domain. These leading countries have contributed to UXS-mHealth apps research and have advanced the domain through local and international partnerships.

#### Leading Research Entities Contributions

Analyzing research entities independently of country affiliation in UXS-mHealth apps publications reveals distinct patterns compared to country-level analysis. Although country-based assessments often emphasize geopolitical trends, research entity analysis provides a more granular view of institutional characteristics. This analysis encompasses 1948 research entities, including academic institutes, hospitals, medical centers, and private organizations. Such diversity suggests the multidisciplinary nature of the field, where theoretical research, practical health care applications, and commercial development contribute to the growth of UXS-mHealth app publications. Table 2 ranks the top 30 research entities based on bibliometric indicators, including TP and citations received. The clustered bar chart compares these metrics for each research entity contributing to UXS-mHealth apps research, which is offered in Multimedia Appendix 3.

**Table 2.** Top 30 research entities, out of 1948, contributed to research on UXS-mHealth apps^a^ from 2007 to 2023. The ranking of the research entities is based on 2 bibliometric indicators: the total publications (TP) and the number of citations received (TC).

Rank	Research entity	Country	TP	TC	TC/TP
1	University of Toronto	Canada	30	373	12.43
2	University College London	The United Kingdom	27	362	13.41
3	University of Sydney	Australia	18	352	19.56
4	University of Melbourne	Australia	17	224	13.18
5	Deakin University	Australia	17	121	7.12
6	University of Utah	The United States	16	453	28.31
7	University of Queensland	Australia	16	234	14.63
8	University of California	The United States	16	160	10.00
9	University of Copenhagen	Denmark	15	113	7.53
10	National University of Singapore	Singapore	15	56	3.73
11	The University of Sydney	Australia	13	100	7.69
12	Sacred Heart University	Italy	13	84	6.46
13	Karolinska Institute	Sweden	12	137	11.42
14	University of Michigan	The United States	12	102	8.50
15	IRCCS Policlinico Gemelli	Italy	12	84	7.00
16	University of California System	The United States	11	462	42.00
17	University Road	Ireland	11	224	20.36
18	University of Pennsylvania	The United States	11	158	14.36
19	University of Washington	The United States	11	142	12.91
20	King’s College London	The United Kingdom	11	126	11.45
21	Zhejiang University	China	11	44	4.00
22	Northwestern University	The United States	10	756	75.60
23	University of California San Francisco	The United States	10	206	20.60
24	University of British Columbia	Canada	10	180	18.00
25	King Saud University	Saudi Arabia	10	170	17.00
26	Monash University	Australia	10	119	11.90
27	Bina Nusantara University	Indonesia	10	31	3.10
28	Imperial College London	The United Kingdom	9	668	74.22
29	Johns Hopkins University	The United States	9	641	71.22
30	University of Pittsburgh	The United States	9	250	27.78

a UXS-mHealth apps: user experience and satisfaction with mobile health apps.

The University of Toronto in Canada leads the list with 30 publications, followed by University College London with 27. Several Australian institutions, including the University of Sydney (n=18), the University of Melbourne (n=17), and Deakin University (n=17), also feature prominently. Notably, research entities from the United States account for 33.33% of the top contributors, whereas Australian institutions contribute 20%. Although Canada has fewer institutions in the top 30, the University of Toronto leads research production in UXS-mHealth apps.

This analysis also uncovers distinct patterns in specialization and influence among the entities. While producing fewer publications, certain institutions demonstrate high citation impact, suggesting groundbreaking contributions to specific subfields. For example, Northwestern University in the United States leads in citations with 756, followed by Imperial College London in the United Kingdom with 668 citations, and Johns Hopkins University in the United States with 641. This highlights that some institutions achieve significant academic impact even with fewer TP, underscoring their role as thought leaders within the field.

#### Distribution of Literature Sources

The analysis of the leading sources for UXS-mHealth apps reveals a total of 351 sources, comprising 260 journals and 91 conferences, demonstrating the extensive research activity in this area. Table 3 depicts that the most prolific journals account for 56.26% (458/814) of the TP in the dataset. Notably, among the most influential sources of knowledge, the *Journal of Medical Internet Research* and its specialized series emerge as critical contributors. Although *JMIR mHealth and uHealth* leads the list with a total of 74 publications and 2512 citations, the journals’ list, including *JMIR Formative Research*, *JMIR Human Factors*, *JMIR Mental Health*, *JMIR Diabetes*, *JMIR Cardio*, and *JMIR Pediatrics and Parenting*, plays a pivotal role in advancing research related to UXS-mHealth apps.

**Table 3.** Top 30 scientific sources contributing to UXS-mHealth apps^a^ publications from 2007 to 2023, showcasing the number of publications (TP) and impact based on total citations received (TC).

Ranking	Source title	TP	TC
1	*JMIR mHealth and uHealth*	74	2512
2	*JMIR Formative Research*	60	234
3	*Journal of Medical Internet Research*	52	2493
4	*JMIR Human Factors*	28	406
5	*Lecture Notes in Computer Science (including subseries Lecture Notes in Artificial Intelligence and Lecture Notes in Bioinformatics)*	25	58
6	*JMIR Research Protocols*	18	165
7	*Digital Health*	17	56
8	*ACM International Conference Proceeding Series*	15	44
9	*International Journal of Medical Informatics*	13	646
10	*International Journal of Environmental Research and Public Health*	12	139
11	*Frontiers in Public Health*	12	86
12	*Telemedicine and e-Health*	11	432
13	*BMC Medical Informatics and Decision Making*	10	171
14	*Studies in Health Technology and Informatics*	10	166
15	*PLoS ONE*	8	398
16	*JMIR Mental Health*	8	153
17	*Journal of Diabetes Science and Technology*	8	126
18	*Internet Interventions*	8	72
19	*BMJ Open*	8	66
20	*Communications in Computer and Information Science*	7	14
21	*IEEE Access*	6	167
22	*JMIR Diabetes*	6	57
23	*JMIR Cardio*	6	38
24	*Advances in Intelligent Systems and Computing*	6	5
25	*BMC Public Health*	5	158
26	*Healthcare (Switzerland)*	5	41
27	*Pervasive Health: Pervasive Computing Technologies for Healthcare*	5	36
28	*International Journal of Human-Computer Interaction*	5	31
29	*JMIR Pediatrics and Parenting*	5	30
30	*Conference on Human Factors in Computing Systems*	5	29

a UXS-mHealth apps: user experience and satisfaction with mobile health apps.

#### Leading Authors’ Contributions

The analysis of the leading authors’ contributions to UXS-mHealth apps highlights a total of 4523 authors who have collectively contributed to the field’s body of knowledge. Table 4 illustrates that the most prolific authors have a concentrated research output. Mohamed Abdelrazek leads the productivity metric with eight publications, followed closely by Liam Glynn, Gearóid ÓLaighin, Leo R. Quinlan, and Richard Harte from the National University of Ireland, Galway, each with 5 publications. Notably, authors affiliated with the National University of Ireland, Galway, play a pivotal role in shaping the discourse surrounding UXS-mHealth apps. A total of 5 of their researchers rank among the top 10 prolific authors, underscoring the institution’s prominence in this research area.

While these authors hold the second spot in publication volume, they achieved the highest impact, amassing 304 citations, which signifies the broad influence and high relevance of their work in the field. In addition, authors from Finland, specifically from the VTT Technical Research Center of Finland, the University of Jyväskylä, and Tampere University, have made significant contributions. Their influence, based on the TC they received, underscores the quality and innovation of their findings in advancing the field.

**Table 4.** The 20 most prolific authors contributed to UXS-mHealth apps^a^ publications from 2007 to 2023, summarizing the number of publications (TP), and impact based on total citations received (TC), country, and affiliation.

Author name	TP	TC	Country	Affiliation
Abdelrazek, Mohamed	8	57	Australia	Deakin University
Glynn, Liam	5	304	Ireland	School of Medicine, National University of Ireland Galway
ÓLaighin, Gearóid	5	304	Ireland	School of Engineering & Informatics and the CÚRAM SFI Center for Research in Medical Devices, National University of Ireland Galway
Quinlan, Leo R.	5	304	Ireland	School of Medicine, and the CÚRAM SFI Center for Research in Medical Devices, National University of Ireland Galway,
Harte, Richard	5	304	Ireland	Human Movement Laboratory and the CÚRAM SFI Center for Research in Medical Devices, National University of Ireland Galway
Mattila, Elina	5	299	Finland	VTT Technical Research Center of Finland
Grundy, John	5	52	Australia	Monash University
Scharf, Thomas	4	289	Ireland	Irish Center for Social Gerontology at the National University of Ireland, Galway
Lappalainen, Raimo	4	219	Finland	University of Jyväskylä
Ahtinen, Aino	4	215	Finland	Tampere University
King, Dominic	4	193	The United Kingdom	Imperial College London
Denecke, Kerstin	4	110	Switzerland	Bern University of Applied Sciences
Schueller, Stephen M.	4	46	The United States	University of California
Bonti, Alessio	4	16	The United States	IBM Research
Lei, Jianbo	4	13	China	Peking University
Wang, Tong	4	13	Japan	Ritsumeikan University
Rodríguez–Molinero, Alejandro	3	275	Spain	Consorci Sanitari del Garraf (CSG)
Orji, Rita	3	263	Canada	Dalhousie University
Alqahtani, Felwah	3	263	Canada	Dalhousie University
Kaipainen, Kirsikka	3	219	Finland	Tampere University

a UXS-mHealth apps: user experience and satisfaction with mobile health apps.

### Bibliographic Science Mapping

#### Citation Analysis

Exploring the annual most cited references is a citation analysis metric. The summary of the most cited publications per year can be found in Multimedia Appendix 4. The exclusion of data from 2007 to 2010 in this table is due to the relatively low number of publications during those years, which makes them less relevant for analyzing annual trends compared to the surge from 2011 onward.

#### Co-Citation Analysis

The co-citation analysis is useful for revealing the most influential publications in a field [69]. This study encompasses 51,612 cited references from 814 publications between 2007 and 2023 on UXS-mHealth apps. We used the CiteSpace (6.4.R1-64-bit-Advanced) co-citation tool default settings to identify and visualize the most co-cited references, highlighting seminal works in the UXS-mHealth apps domain.

The co-citation cluster and the strongest citation bursts are presented in Multimedia Appendix 5. The major co-citation clusters, labeled in gray, where the cluster number indicates the cluster volume. The smaller the number, the larger the volume. Clusters such as 1, 9, and others that are unrepresented in the network suggest that they are large but isolated outside the main network structure. The color of each cluster reflects the average publication year, with greenish clusters representing earlier works from 2013 to 2016 and yellow clusters signifying more recent publications from 2018 to 2020. The number of nodes in each cluster corresponds to the volume of publications; larger nodes indicate greater connectivity. Notably, the largest node in each cluster represents a highly influential publication, demonstrating its prominence within that area. In addition, the analysis highlights the top 8 publications with the strongest citation bursts. This indicates periods of intense scholarly attention and influence within the research domain. Notably, the study “Mobile App Rating Scale: A New Tool for Assessing the Quality of Health Mobile Apps” by [86] leads the list, reflecting its significant impact and influence in the field.

The synthesized findings from the 8 studies provide a comprehensive understanding of various aspects of the use, design, and evaluation of mHealth apps, focusing on quality assessment, user engagement, usability, and retention. Each study offers unique insights; however, in combination, they present a cohesive overview of key factors that influence the adoption and effectiveness of mHealth technologies.

One of the primary areas of focus is the quality assessment and rating of mHealth apps. Researchers have developed comprehensive tools to address the limitations of traditional star-based rating systems. For example, the Mobile App Rating Scale (MARS) was introduced as a 23-item tool that evaluates apps across 5 categories: engagement, functionality, esthetics, information quality, and subjective quality. The proposed tool demonstrated excellent psychometric properties, making it a reliable framework for app classification and quality assessment [86]. Complementing this effort, the Mobile Health App Usability Questionnaire (MAUQ) was created to address usability gaps in existing tools, such as the System Usability Scale (SUS) and the Post-Study System Usability Questionnaire (PSSUQ) [87]. The MAUQ was tailored for interactive and standalone apps and demonstrated strong reliability and validity. The MARS and MAUQ are foundational instruments for assessing app quality and usability, offering structured approaches for researchers and developers [86,87].

The publications on user engagement emphasize the crucial role that effective design and functionality play in maintaining app usage. The study explored how users interact with health apps, revealing that initial engagement often declines after reaching certain milestones. Factors such as app design, ease of use, and data management significantly influence this decline [74]. To address this problem, “effective engagement” was proposed, indicating that sufficient interaction with an app is necessary to achieve the desired health outcomes. Tailored interventions that consider individual user needs and contexts are recommended to enhance engagement [88]. A focused study on mental health apps identified barriers to engagement, including a lack of user-centered design and privacy concerns. The findings emphasized the necessity for a user-centric approach to improve sustained engagement and adoption [89].

Usability is critical in influencing user satisfaction and the long-term use of mHealth apps. A comprehensive review of usability testing methods highlighted a predominance of traditional techniques, such as questionnaires, interviews, and task completion protocols [90]. Although questionnaires provide broad usability insights, qualitative methods like “Think-Aloud” protocols are more effective in pinpointing specific usability issues [90]. In addition, there remains a significant underuse of automated testing methods, such as eye-tracking, which can provide objective usability insights [90]. These findings align with the emphasis on developing robust usability instruments like MAUQ, which can adapt to different app types and user contexts [87]. These studies indicate that combining qualitative and quantitative methods is essential for a holistic understanding of usability, and further research should explore integrating automated testing tools to streamline the evaluation process.

Postadoption behavior and retention are critical for the long-term success of health apps. Researchers used models, such as the Post-Acceptance Model and Technology Acceptance Model (TAM), to examine factors influencing the continued use of health apps. They found that perceived usefulness, ease of use, confirmation, and satisfaction significantly predict the intention to continue using the app [91]. Consistent with these findings, an analysis of real-world usage patterns of mental health apps revealed that only a small proportion of users maintain long-term engagement [92]. Certain app features, such as mindfulness and peer support, were associated with higher retention rates than other functionalities, such as trackers or breathing exercises [92]. These results suggest that long-term retention is driven by the perceived value and satisfaction derived from specific app features rather than the overall app experience [91,92].

#### Coauthorship Analysis

The coauthorship analysis aimed to explore the social structure and collaborative networks among researchers contributing to the field of study [69]. VOSviewer (version 1.6.20) was used for this analysis, and the “authors” were selected as the unit of analysis. The UXS-mHealth research dataset comprised 814 publications from 2007 to 2023, with contributions from 4870 unique authors. A sensitivity analysis was conducted on the coauthorship network, focusing on 3 attributes: documents, citations, and total link strength. Based on this analysis, a threshold of three documents per author was established to ensure meaningful representation, and 158 authors met the criteria. To enhance the coauthorship network visualization, the coauthorship network illustrates collaborations among the top 100 authors.

The resulting coauthorship network depicts 19 distinct clusters, each distinguished by a unique color. Larger node sizes indicate the most significant collaborative relationships based on the total number of citations received. The coauthorship collaborations among the top 5 cited clusters reveal knowledge-sharing patterns and intellectual partnerships within the UXS-mHealth research. Furthermore, using the CiteSpace tool, the co-citation analysis for authors reveals the most cited authors, their citation strength, and citation bursts over the years. The coauthorship networks, the coauthorship theme summary, and the coauthor citation bursts can be found in Multimedia Appendix 6.

#### Co-Word Analysis

Co-word analysis investigates the relationships between topics by focusing on the content of publications rather than their bibliographical details [69]. This approach allows for a deeper understanding of existing or potential connections among themes within the research. Co-word analysis examines existing or future relationships among topics because it targets publications’ content rather than bibliographical units [69]. In this study, co-word analysis is used to examine 2210 author keywords used in 814 UXS-mHealth apps publications from 2007 to 2023.

**Figure 5.**
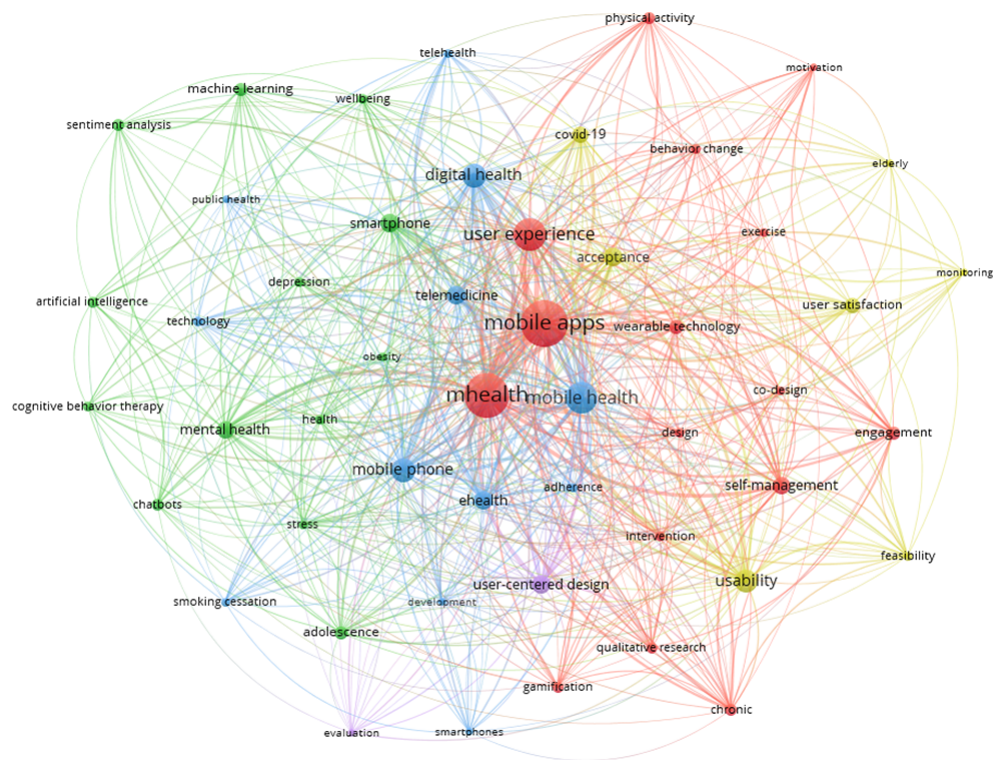
The co-word analysis of author keywords for user experience and satisfaction with mobile health apps from 2007 to 2023 identified the top 50 keywords co-occurring within 6 distinct clusters.

**Figure 6.**
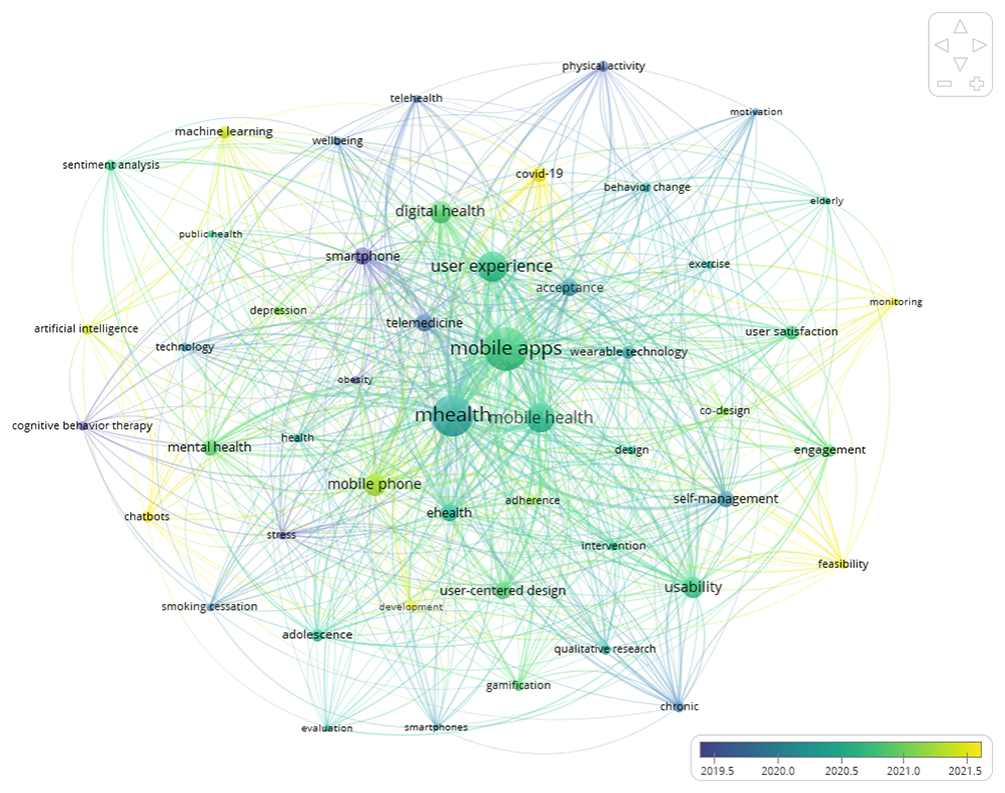
The co-word analysis for the author keyword on user experience and satisfaction with mobile health apps is represented in the timeline network for the top 50 keyword co-occurrences.

The tool used to create the co-word network is VOSviewer (version 1.6.20) for author keywords as a unit for co-occurrence analysis. The keywords that meet a threshold of at least three occurrences are 403. The 50 most frequently occurring keywords are selected for network visualization. The network visualization captures their relationships, with node size representing frequency and link thickness indicating co-occurrence frequency within six distinct clusters depicted in Figure 5. In addition, Figure 6 visualizes the author keywords co-occurrence network timeline, illustrating the dynamic evolution of research themes in UXS-mHealth apps over time. For frequency analysis, Multimedia Appendix 7 includes the rank for the 30 most frequent keywords.

This timeline shows when certain keywords began gaining prominence and whether their popularity has grown or diminished. For instance, keywords such as artificial intelligence, machine learning, chatbots, and feasibility may exhibit a steep rise in frequency from 2021, indicating emerging topics that are receiving increased attention from the mHealth research community. In contrast, other keywords, such as telemedicine, may show a decline, suggesting that interest in related topics has waned. For clustering analysis, the network of keywords can be further broken down into thematic clusters. These clusters reveal how topics are grouped and provide insights into the thematic structure of the research field. Figure 5 depicts how the red and blue clusters are tightly connected, indicating specialized areas of study, while others display overlapping themes, such as the purple cluster, suggesting interdisciplinary efforts. Each cluster represents a specific content hotspot within the domain, providing a snapshot of the diverse areas being explored. Overall, the co-word analysis reveals that the six identified clusters provide insights into how digital health technologies are conceptualized, developed, and implemented to address different health needs.

#### Bibliographic Coupling

**Figure 7.**
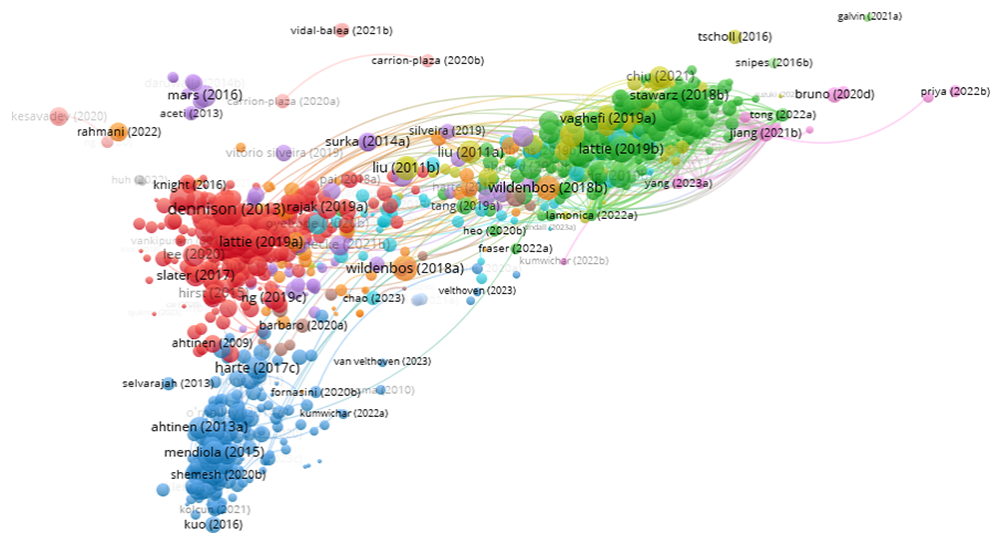
The bibliographic coupling network of 813 publications on user experience and satisfaction with mobile health apps from 2007 to 2023 identifies 18 clusters. Each color represents a cluster, while the node’s size represents the total number of citations a publication received. The order of the most significant clusters based on total publications is (red, green, blue, yellow, and purple).

Bibliographic coupling is a science mapping technique to analyze the relationships among citing publications, assuming 2 publications can be similar if they share references, enabling the grouping of publications into thematic clusters [69]. In this study, the bibliographic coupling analysis conducted on 814 publications for the UXS-mHealth apps from 2007 to 2023 reveals 18 distinct clusters, each representing a group of publications sharing commonly cited references as depicted in Figure 7. The tool used to create the bibliographic coupling network is VOSviewer (version 1.6.20), which is used to analyze bibliographic coupling at the document level, for the full counting method, and for a minimum of zero citations of a document. Normalization was performed using the association strength method. The layout was generated with default values (attraction=2 and repulsion=1). Clustering was conducted with a resolution of 1.00, a minimum cluster size of 1, and with the option to merge small clusters enabled. Among these are the largest clusters (341 publications in red color), followed by the green cluster (242 publications), the blue cluster (218 publications) grouped under the label Patient Engagement, Usability, and Design, the yellow cluster (71 publications), and the purple cluster (56 publications).

The tool used to create network visualization reveals cluster overlap, indicating potential thematic intersections or conceptual similarities across different research streams. This overlapping may arise due to shared foundational literature, similar methodological approaches, or overlapping research topics examined from different disciplinary perspectives. However, the size and structure of these clusters indicate varying degrees of thematic similarity among the grouped publications, suggesting the concentration of research activity in specific thematic areas.

**Figure 8.**
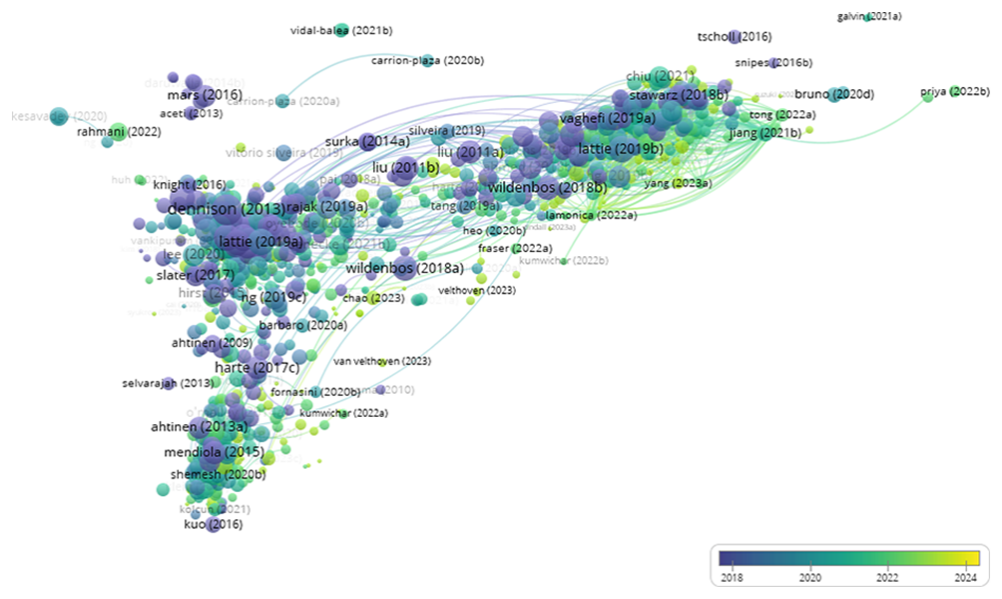
The bibliographic coupling timeline network of 813 publications on user experience and satisfaction with mobile health apps from 2007 to 2023, showing clusters 2 and 4, includes more recent publications than others.

These 5 significant clusters are further analyzed by reviewing the titles, abstracts, and keywords of the top 10 most cited studies in each cluster to uncover the thematic development within each research group. Figure 8 shows the bibliographic coupling timeline network based on the publication year, where clusters 2 and 4 include more recent publications. While Table 5 summarizes clusters of information.

**Table 5.** The descriptive summary of the most significant clusters includes total publications (TP), cluster time (years), the top citation and publication year, and the suggested theme labels.

Cluster	Theme	TP	Years	Top citation (year)
1	Evaluating technological elements in using mHealth^a^ apps and wearables	341	2008‐2023	623 (2013)
2	Key factors influencing user engagement and experience or app design features enhancing user engagement	242	2013‐2023	355 (2019)
3	Evaluation of mHealth app effectiveness and acceptance for patient self-management	218	2010‐2023	190 (2017)
4	Determinants of mHealth user experience and long-term engagement	75	2011‐2023	230 (2015)
5	Barriers and enablers of mHealth integration in clinical practice	53	2011‐2023	131 (2016)

a mHealth: mobile health.

### Systematic Literature Review

Through a systematic procedure, keyword and thematic analyses were performed. The keyword analysis included the analysis of the co-occurrence of 403 keywords at least 3 times. Keyword analysis provides insights into the most frequently used terms in titles, abstracts, and keywords, thereby offering a high-level view of recurring topics and areas of interest [69]. In addition, thematic analysis based on bibliographic coupling includes 928 articles to further explore the underlying themes and patterns within the research, revealing the conceptual frameworks and areas of scholarly focus [69]. By combining these methods, the following section discusses evolving research trends, thematic analyses, dominant theories, and areas requiring further exploration.

## Discussion

### Research Trend Analysis

A total of 2 research trends in UXS-mHealth apps are presented: differentiation in using the keywords “user experience” and “user satisfaction” and use of research methodologies.

#### User Experience and User Satisfaction

User experience and user satisfaction were among the most frequently occurring keywords, reflecting the initial search scope of this study. User experience appeared 218 times with 47 links and a total link strength of 564, whereas user satisfaction had 49 occurrences, 22 links, and a total link strength of 104 (see Figure 5 and Multimedia Appendix 7). These keywords belonged to different clusters despite their conceptual similarities, indicating potential research gaps. Furthermore, user experience focused on themes such as behavioral change, engagement, and interventions, emphasizing sustained interaction via design features. Associated keywords such as “behavior change,” “gamification,” and “mobile apps” highlighted their role in influencing long-term behaviors and improving health outcomes. In contrast, user satisfaction was related to the short-term evaluations of functionality and usability, with keywords such as “acceptance,” “usability,” and “monitoring” reflecting a focus on meeting immediate user expectations. User satisfaction is more transactional and often measured via usability testing or acceptance in specific contexts such as “COVID-19” or “elderly” populations. The weak co-occurrence link between these concepts indicated a gap in the research on mHealth apps and that the efforts used to enhance user experience were not systematically aligned with strategies to improve user satisfaction. This disconnect indicated an opportunity for further research to explore how better design can drive both user engagement and user satisfaction.

#### Theory-Driven Versus Data-Driven Research

The analysis of keywords and themes for the research on UXS-mHealth apps from 2017 to 2013 revealed that 32.2% (262/813) of publications included the terms “theor*,” “model,” or “survey” in their title, keywords, or abstract, as summarized in Table S1 in Multimedia Appendix 8. Among these studies, the TAM, health belief model (HBM), and Unified Theory of Acceptance and Use of Technology (UTAUT) were most frequently used in 38.9% (102/262), 11.5% (30/262), and 9.5% (25/262) of studies, respectively. The top 10 most cited studies [16,17,74,93-99] that applied the theoretical framework were also reviewed to explore their shared research theme.

These studies primarily focused on the use of mHealth technologies for health monitoring, self-management, and behavior change, particularly for chronic conditions and mental health. They assessed the usability, functionality, and effectiveness of mHealth apps for engaging users in self-care, improving health outcomes, and promoting sustained usage. They applied behavioral frameworks to explore factors influencing user experience, including technical functionality, engagement features, ease of use, and user satisfaction, aiming to inform the design and optimization of health apps that effectively meet user needs. A shared goal of these studies was to leverage theoretical grounds for evaluating mHealth technologies to create scalable, accessible, and evidence-based mHealth interventions that enhance health monitoring and behavior change.

The timeline network in Figure 6 shows that the keywords “artificial intelligence,” “machine learning,” and “sentiment analysis” lead the recent keywords list. Thematic analysis also revealed the same results, where data-driven research was conducted using machine learning, artificial intelligence, and sentiment analysis. The analysis of titles, abstracts, and keywords of the data corpus revealed that research on UXS-mHealth apps began incorporating machine learning in 2017, sentiment analysis in 2018, and artificial intelligence in 2019, as indicated by the initial publication years see Table S2 in Multimedia Appendix 8). The terms “machine learning,” “sentiment analysis,” and “artificial intelligence” were used in 4.18% (34/814), 5.17% (42/814), and 3.08% (25/814) of studies, respectively. The most frequently cited studies related to the terms “machine learning,” “sentiment analysis,” and “artificial intelligence” are [13 ,100], [101,102], and [103,104], respectively.

To understand the trends in these studies, we reviewed the most cited studies that explored machine learning, sentiment analysis, or artificial intelligence to address various challenges with mHealth apps, particularly those focused on mental health, physical activity, and chronic disease management. In particular, we focused on studies that evaluated and enhanced health-related apps and wearable devices to improve user experience and treatment outcomes. For instance, machine learning and thematic analysis were used to identify barriers and enablers that impacted the effectiveness of mental health apps [105]. Chatbots apply cognitive behavioral therapy principles to support emotional regulation and behavior change in adolescents managing obesity and prediabetes. The research on activity trackers examined predictors of sustained use and behavior modification, highlighting technical issues and user experience as critical determinants. Similarly, the development of automated cough detection systems underscored the potential for artificial intelligence–powered tools to facilitate continuous, objective health monitoring. These studies highlighted the potential of analyzing user feedback and leveraging sentiment analysis and artificial intelligence to enhance health outcomes. By making mHealth apps more engaging, accessible, and responsive to user needs, these approaches can help develop more scalable, cost-effective solutions that address mHealth challenges.

The theory-driven versus data-driven research trend revealed that future studies should continue using a mixed-method approach that combines the strengths of both paradigms. Integrating theoretical and qualitative approaches with data-driven, quantitative methodologies enables a multidimensional analysis of UXS in mHealth applications, fostering a holistic understanding of their design and impact. Furthermore, theoretical insights contribute depth and context, whereas data-driven results offer generalizability and objectivity. This combination can enhance the robustness of findings and facilitate impactful, multifaceted conclusions that bridge gaps across diverse perspectives in the UXS-mHealth apps field while potentially increasing relevance and applicability in future research.

The observed trends also highlight the need for a new methodological triangulation approach that combines the strengths of both paradigms. By integrating quantitative insights with data-driven, qualitative analysis, particularly via text analytics and machine learning algorithms on user reviews, researchers can comprehensively understand the challenges associated with UXS in mHealth apps. Quantitative perspectives provide structured insights, whereas qualitative data from user reviews add depth and context to the results, uncovering patterns and sentiments that might otherwise remain hidden. This methodological integration will strengthen the robustness of findings and support the development of impactful and layered conclusions, thereby connecting diverse perspectives within UXS-mHealth research. Such an approach promises to enhance the relevance and practical applicability of future studies in this field.

### Research Thematic Analysis

The 5 largest thematic clusters obtained from the bibliographic analysis included in Table 5 are discussed in the following section. The studies that overlapped across themes were assigned to all relevant themes. The keyword analysis for each cluster is included in Multimedia Appendix 9.

#### Theme 1: Evaluating Technological Elements in Using mHealth Apps and Wearables

This theme was the largest cluster identified during bibliographic coupling and focused on reviewing technological elements in mHealth apps and wearables designed for monitoring health behavior change, offering mental health support, and physical activity tracking. This cluster evaluated the key factors influencing user engagement, usability, and long-term use of mHealth apps, particularly those designed for mental health, fitness, and self-management purposes. A significant number of studies within this cluster conducted SLRs to confirm findings from existing research and assess the effectiveness of mHealth apps and user satisfaction.

Various methods were employed to explore users’ perspectives on mHealth apps and wearables related to behavior change. Qualitative approaches dominated in these studies, and systematic reviews [12,15,19,50,106], interviews [75], and focus groups [107] were the most frequently used techniques. This cluster also comprised studies that used quantitative methods such as surveys [98] and randomized controlled trials [106].

Theoretical frameworks further enriched the analysis, providing a foundation for understanding user interaction with mHealth apps. The research was structured using a combination of TAM, the health information technology acceptance model (HITAM) [74], and UTAUT [98], as well as a combination of the analytical hierarchy process (AHP) and Fuzzy Technique for Order Preference by Similarity to Ideal Solution (Fuzzy TOPSIS) [16].

The corresponding findings were published in several prominent journals in health technology, medical informatics, and social sciences, including *JMIR mHealth and uHealth*, *Journal of Medical Internet Research*, *PLoS ONE*, *Telemedicine and e-Health*, *Journal of Cardiovascular Nursing*, *Technology in Society*, and *Psychiatric Services*.

Key factors affecting the success of mHealth apps and wearables emerged from several influential studies in this cluster. For instance, app accuracy, security, and efforts required for use were the significant determinants of user engagement [107]. In addition, performance expectancy, hedonic motivations, and price value were the predictors of continued use [98]. The ability of the apps and wearables to track behavior and goals, along with the option to receive advice and information quality, was highly valued by users [16,107]. Other important factors, such as interface design, navigation, notifications, and actionable recommendations, were critical in influencing user engagement [75].

Despite the opportunities created by advances in mHealth technologies, studies in this cluster highlighted several challenges [12,15,74,107]. The majority of studies agreed on the existence of a significant research gap regarding the validation of elements that promoted long-term app use and engagement [12,15,19,50,74,106]. These studies also highlighted the variability in evaluating mHealth apps and wearables, necessitating the development of standardized measures to assess the effectiveness factors on UXS, usability, and engagement [15,16,50].

Future recommendations include exploring hybrid trial designs, developing user-centered designs, and addressing the long-term efficacy and scalability of mHealth interventions [55,57,58]. Overall, these studies highlighted the opportunities and challenges associated with mHealth apps, particularly in sustaining user engagement, improving usability, and ensuring the long-term effectiveness of health behavior change interventions [54,55,58]. Promising future research directions include the development of more robust frameworks for evaluating the long-term impact of these technologies on health outcomes and user behavior. The integration of emerging technologies such as artificial intelligence and machine learning into mHealth solutions presents new opportunities for enhancing personalization and improving sustained engagement.

#### Theme 2: Key Factors Influencing User Engagement and User Experience or App Design Features Enhancing User Engagement

Thematic synthesis of studies highlighted shared objectives, methodologies, and findings that focused on improving mHealth across various domains, particularly mental health and chronic disease management. A recurring aim in these studies was to explore the effectiveness, user engagement, and design features of mHealth apps, focusing on enhancing user experience (UX) and sustained use. For instance, mental health apps of [15,17-19], medication adherence [12], physical activity apps [13], and chronic disease management [6,14] were focused on in the cited studies. The publication sources used for this cluster were drawn from leading academic journals and conferences that offered diverse and valuable perspectives on mHealth research. These included *JMIR mHealth and uHealth*, *Diagnostics*, *The International Journal of Engineering and Technology (UAE)*, and the 7th International Conference on I-SMAC (IoT in Social, Mobile, Analytics, and Cloud).

Most studies in this cluster predominantly used SLR to evaluate various types of mHealth apps, ranging from mental health and medication adherence to chronic disease management [6,12,15,19]. mHealth apps were reviewed to thematically analyze their features [12,16], and qualitative thematic analysis was performed to analyze app user reviews and extract engagement features [18]. Quantitative data [13] collected via a questionnaire and an activity tracker were analyzed using random forest—a machine learning technique—to identify the determinants of long-term engagement. Theoretical frameworks were only represented by Jeffrey et al [14] using semistructured interviews based on the TAM and HITAM to assess the factors that influenced the use of diabetes apps and [16]; they also used the AHP and fuzzy TOPSIS framework to evaluate mHealth apps.

Findings across these studies consistently revealed that factors such as usability, personalization, and user satisfaction were critical for the success of mHealth apps. Rajak et al and Hermsen et al [13,16] emphasized the significance of evaluating mHealth apps using multicriteria decision-making frameworks and machine learning techniques to account for diverse user needs and preferences. Their studies collectively advocated for ongoing research to further refine app functionalities, with a particular focus on their real-world implementation and obtaining continuous user feedback to enhance the overall effectiveness and engagement of mHealth interventions [18,108].

Across these studies, common concepts emerged regarding the importance of specific app features such as reminders and educational content to support user adherence [12]. The role of user satisfaction, ease of use, and user interface design was emphasized by Rajak et al and Alqahtani et al [16,18], who found that customizable, user-friendly apps with varied content and functionalities considerably enhance engagement. In contrast, poor usability and lack of personalization were frequently cited reasons for app abandonment. Adaptable design features tailored to diverse user needs were reported as a crucial factor for maintaining a positive UX [17]. In the context of chronic disease management, users favored apps with educational content, monitoring, and tracking functionalities [6,14]; the critical determinants of sustained engagement included age, goal setting, and UX [13]. A key finding across the studies was that design features such as personalization, reinforcement, and compelling message presentation were vital for maintaining user engagement [8,18].

Although previous studies have identified key design features in mHealth apps, more rigorous, evidence-based research is required to validate the effectiveness of these features. These studies [12,15,19] emphasized the importance of establishing standardized metrics for evaluating user engagement and app efficacy to improve overall user satisfaction. Stawarz et al [17] advocated for developing evidence-based approaches to improve UX in mental health apps. In contrast, Hermsen et al [13] underscored the critical need to focus on technical performance and UX in activity tracking technologies. Alessa et al [6] called for large-scale clinical studies to assess the impact of mHealth apps on chronic disease management, particularly regarding their potential to reduce blood pressure. In addition, Wei et al [8] highlighted the need for more robust quantitative assessments to better understand the correlation between app design features and sustained user engagement.

In summary, future research should prioritize longitudinal studies that examine the impact of personalized and adaptive design features on user retention, health outcomes, and behavioral changes across diverse user demographics. In addition, interdisciplinary research that integrates fields such as behavioral science, human-computer interaction, and health care must be conducted to develop a comprehensive framework for mHealth app design. By investigating the potential of continuously incorporating adaptive machine learning algorithms to tailor app features to individual user preferences and needs, critical insights into optimizing UX can be obtained. Furthermore, research exploring the scalability of mHealth apps in underrepresented or underserved populations can further contribute to the development of inclusive, effective digital health solutions.

#### Theme 3: Evaluation of mHealth App Effectiveness and Acceptance for Patient Self-Management

The analysis of studies focusing on the usefulness and effectiveness of mobile apps for patient management or chronic disease monitoring revealed a diverse set of themes and influencing factors. Researchers primarily used methodologies such as qualitative interviews, systematic and narrative reviews, thematic analysis, and quantitative techniques such as questionnaires, regression analyses, and randomized control trials to evaluate mHealth apps in the literature reviewed.

These studies were published in health research journals, each with a different perspective. *JMIR mHealth and uHealth* focus on patient-centric mHealth apps and technology usability. Journal of Medical Internet Research offers a broad, interdisciplinary approach to eHealth, emphasizing theoretical and clinical apps. *JMIR Research Protocols* are methodologically oriented, highlighting research transparency and design considerations. *JAMA Internal Medicine* provides a clinical and health policy perspective, prioritizing robust evidence for clinical effectiveness and health outcomes. Each journal contributes uniquely to understanding mobile health interventions, reflecting their specific editorial focus and research priorities.

The key themes identified from the reviewed studies can be broadly categorized into technical, health-related, and usability factors. Mendiola et al identified five core factors affecting the performance of mHealth apps: plan or order, data export, usability, cost, and tracking features [4]. Similarly, design implications that focused on enhancing everyday life, providing user flexibility, and supporting self-improvement were emphasized [5]. Wei et al synthesized findings from 35 studies and categorized critical success factors into personalization, reinforcement, communication, navigation, credibility, message presentation, and interface esthetics [8]. The standardized software usability measurement inventory that uncovers five user satisfaction dimensions, that is, efficiency, effectiveness, helpfulness, controllability, and learnability, was applied [9]. Other studies expanded the thematic scope to include technical (eg, ease of use and data-related issues), health-related (eg, integration into the patient journey and insurance status), and social factors (eg, demographic and cultural aspects) [62]. Crafoord et al [109] explored patient engagement via symptom reporting and highlighted the need for continuous feedback and comprehensive information to support patients throughout their treatment journey.

Several studies mentioned patient feedback and continuous support [63]; however, only a few studies have addressed how mHealth apps fully integrate with patients’ broader healthcare journeys, particularly regarding long-term adherence and coordination with healthcare providers. The influence of demographic and cultural factors [62] remains underexplored, particularly in their effect on the usability and acceptance of mHealth apps across diverse populations. Future research should address such gaps by focusing on the influence of cultural and demographic diversity on app design and user satisfaction, ensuring personalization in mHealth innovations.

Mendiola et al and Wei et al [4,8] explored technical aspects such as data export and navigation; however, data privacy and security remain unexplored. Moreover, the influence of these factors on user trust and satisfaction, which are critical concerns in the modern healthcare technology landscape, has not been sufficiently studied. This gap should be addressed in future research by focusing on trust and data security concerns, which are becoming increasingly critical as healthcare apps become more complex and data-driven. Understanding how to build and maintain user trust while ensuring robust data security measures will be essential for successfully adopting and using these apps [6].

#### Theme 4: Determinants of mHealth UX and Long-Term Engagement

The studies within this cluster collectively explored various factors that influenced adoption, UX, and long-term engagement with mHealth apps. These determinants shape the perception of users regarding the utility of mHealth apps and their intention to continue using them. Common concepts identified include user satisfaction, perceived usefulness, personalization, technical functionality, and social influence. A critical finding across several studies was the importance of user satisfaction in driving the continuous use of mHealth apps. Liu et al [10] demonstrated the impact of unique smartphone features, such as context awareness and portability, on enhancing user satisfaction by improving app functionality. Similarly, Vaghefi and Tulu [75] underscored that UX is pivotal for maintaining user engagement, particularly in interface design and navigation. Birkmeyer et al [110] highlighted how personalization, social networking, and app design contribute to user satisfaction, directly correlating with continuance intentions and word-of-mouth recommendations.

Furthermore, perceived usefulness was a recurring determinant of whether users will continue to engage with mHealth apps. Factors such as performance expectancy and the confirmation of expectations consistently emerged as drivers of sustained app use. Chiu et al [94] emphasized that emotional and temporal investments in an app contribute to a sense of commitment and encourage its continuous usage among users. Chiu et al and Yuan et al [94,98] found that users’ beliefs regarding the utility of health apps considerably impacted their behavioral intentions. Personalization was another central theme that influenced long-term engagement with mHealth apps in their studies. Biduski et al and Lappalainen et al [20,111] highlighted how personalized features, such as goal setting, tailored feedback, and user-system fit, are crucial for retaining users over time. They also reported that customizing app functionalities to meet individual needs fostered a sense of relevance and enhances user engagement. Al Ayubi et al [112] suggested that offering diverse physical activities within mHealth apps increased user motivation by aligning the content with their specific health objectives.

Several studies identified technical challenges and usability issues as barriers to long-term app use. Biduski et al [20] emphasized that software glitches and repetitive data-entry processes diminished user motivation. Ehn et al [113] observed similar challenges, noting that older adults struggled with the complexity of activity monitors that was exacerbated by a nonintuitive design. These usability issues must be addressed by simplifying interfaces and improving technical reliability to maintain user engagement, particularly among less tech-savvy populations.

The social dimension of mHealth app use still plays a role in shaping user behavior. Al Ayubi et al [112] demonstrated that social networking features motivated users to engage in physical activity through peer support and competition. However, Yuan et al [98] found that social influence posed minimal impact on the continued use of health apps, suggesting that different social norms may affect users differently. Future research can investigate these nuances to better understand the contribution of social factors to sustained app usage.

The studies in this cluster employed diverse research methodologies to explore various factors that influenced user engagement, satisfaction, and long-term use of mHealth apps. Quantitative surveys, grounded in theoretical models such as UTAUT2 and expectation–confirmation model, were used to analyze factors such as performance expectancy, perceived usefulness, and continuance intention; these surveys provided generalizable insights into user behavior [94,98]. In-depth qualitative interviews were also conducted to capture detailed user experiences, motivations, and barriers [75,113], which allowed for a more nuanced understanding of individual behaviors. Several studies [110] employed mixed-method approaches combining quantitative data with qualitative insights to offer a more holistic perspective on the use of mHealth apps. Longitudinal studies [75] tracked user engagement over time, providing valuable insights into the evolution of app usage. Biduski et al [20] introduced an innovative assessment method using in-app embedded conversation-based questionnaires, enabling real-time data collection on UX throughout different stages of app interaction. The methodologies utilized across the studies in this cluster reflected the complexity of investigating user engagement with mHealth apps. Quantitative surveys provided generalizable findings on user behavior, whereas qualitative interviews offered deeper insights into individual experiences. Mixed method approaches and longitudinal studies added further depth by tracking the evolution of user engagement. Together, these methodologies provided a comprehensive understanding of the determinants influencing long-term success of mHealth apps.

The studies in this cluster were published in various journals and cover different aspects of mHealth research, including technical and behavioral areas. For instance, the *Journal of Systems and Software* focuses on the technical foundations of mHealth app development, emphasizing innovations in software design and systems engineering. Telemedicine and eHealth specialize in integrating telecommunication technologies with health care delivery, particularly in underserved areas. *JMIR mHealth and uHealth*—one of the leading journals in this field—frequently publishes studies on app usability, user engagement, and health outcomes. Other notable journals, such as *Information Technology & People*, explore the socio-technical dynamics of mHealth apps. *Computers in Human Behavior* focuses on the impact of technology on user behavior.

#### Theme 5: Barriers and Enablers of mHealth Integration in Clinical Practice

Thematic analysis of 10 most cited publications in this cluster highlighted several critical factors affecting the successful adoption and integration of mHealth apps in clinical settings. Usability and design emerged as consistent themes, with studies identifying poor user interfaces and complexity as significant barriers to the adoption of mHealth apps across various contexts, from chronic disease management to surgical wound monitoring. Multiple studies highlighted the need for seamless integration into existing clinical workflows and identified challenges in workflow disruption, duplication of tasks, and time constraints faced by healthcare professionals. Concerns over privacy and security also featured prominently, where secure data transmission and regulatory compliance appeared as crucial factors for user confidence. Future work in this area should devise guidelines, improve app design, and provide tailored training modules to address the diverse needs of both healthcare providers and patients while ensuring that mHealth apps are adaptable, reliable, and accessible across different technological and cultural contexts.

The analysis further revealed similarities and differences across the research methods and samples used in these studies and the types of journals in which they were published. Research methods varied from literature reviews [114,115] and qualitative evaluations [116] to usability testing [117], mixed methods studies [118,119], focused groups [120], and surveys [121-123]. The samples also differed considerably across studies. Most studies in this cluster focused on involving users or physicians in the development and testing of mHealth apps, with an emphasis on creating user-centered designs [116,118-123].

The focus areas of journals in which these studies were published also differed. *JMIR mHealth and uHealth* and the *Journal of Medical Internet Research* frequently publish studies focusing on mHealth technologies, patient engagement, and telehealth innovations. *BMC Medical Informatics and Decision Making* and the *International Journal of Medical Informatics* cater to broader medical informatics and decision-making processes in clinical settings. *Studies in Health Technology and Informatics* are more focused on advancing health technology, which might offer a more technology-centered lens than patient-focused journals such as *Healthcare*. These differences in journal focus reflect the varied perspectives on mHealth adoption—from usability and design to workflow integration and patient-provider relationships, emphasizing the need for interdisciplinary research approaches in advancing mHealth technologies.

### Limitations

The bibliometric analysis provides a structured overview of the research landscape, and the SLR offers deeper insights into research trends and knowledge gaps, guiding future research directions by combining breadth with depth. However, their limitations are particularly notable in dynamic and interdisciplinary fields like mHealth apps, where technology and knowledge evolve rapidly. Bibliometric analysis may struggle to capture emerging studies, while SLRs are restricted by the preselected sample of articles defined by inclusion and exclusion criteria, which can overlook relevant contributions outside those boundaries. These constraints highlight the importance of interpreting results with caution and updating analyses regularly to ensure relevance. Furthermore, while most of the studies might originate from countries with well-developed digital health infrastructures, they reflect the current global distribution of research and investment. This may limit the generalizability of our findings to underresourced settings, where barriers such as limited internet access, lower digital literacy, and weaker health system capacities are more prevalent. Further research from developing countries is needed to provide a more comprehensive and balanced understanding of digital health implementation.

### Conclusion

This study represents the first bibliometric analysis of user experience and satisfaction with mHealth apps (UXS-mHealth apps). Using a comprehensive data corpus sourced from Scopus and Web of Science, we systematically analyzed the literature through bibliometric performance analysis and science mapping techniques. Our methodology used a variety of data processing and visualization tools, including VOS Viewer, Excel, OriginLab, and SiteSpace, to identify influential publications, authors, countries, and research sources.

The analysis highlights the evolution and emergence of key research topics in this domain by examining annual research trends, co-citation analysis, co-word analysis, and bibliographic coupling. Over a 16-year period (2007‐2023), we analyzed 814 unique publications authored by 4870 researchers from 81 countries and 1948 organizations, published across 351 high-impact journals and prominent conferences. This study provides a foundational understanding of the bibliographic landscape in UXS-mHealth apps by identifying major research clusters, influential works, and emerging topics, providing evidence-based guidance for researchers, developers, and health informatics practitioners.

Furthermore, research trends and themes in UXS-mHealth were systematically reviewed using co-word analysis and bibliographic coupling. This approach provided a comprehensive overview of the existing literature by addressing the limitations of previous studies, which often focused on specific applications, limited time frames, or restricted database coverage. Results revealed key thematic areas, emerging trends, and gaps in mHealth UX research, highlighting the interdisciplinary nature of the field and the need for broader methodological integration. The analysis of research trends revealed 2 trends: the differentiation in using user experience and user satisfaction keywords and the research methodology used in the domain, highlighting the need to incorporate user-generated data into mixed-method research.

Thematic analysis was performed across five most significant themes: (Theme 1) evaluating technological elements in using mHealth apps and wearables, (Theme 2) key factors influencing user engagement and experience or app design features enhancing user engagement, (Theme 3) evaluation of mHealth app effectiveness and acceptance for patient self-management, (Theme 4) determinants of mHealth UX and long-term engagement, and (Theme 5) barriers and enablers of mHealth integration in clinical practice.

The research trends and 5 themes identified in this review explored critical aspects of technology use, user engagement, and clinical integration. While all five themes overlapped, particularly themes 1, 2, and 4, each of them focuses on distinct elements that help delineate their contributions to the overall understanding of mHealth apps: technological evaluation (Theme 1), design features for engagement (Theme 2), patient usability (Theme 3), long-term engagement factors (Theme 4), and clinical integration (Theme 5). By analyzing the identified trends and themes, valuable insights into future research on UXS-mHealth can be gained.

The findings suggest the importance of prioritizing user-centered design and implementing a more engaging experience for mHealth apps. Furthermore, future research should prioritize expanding the scope of UX evaluation by incorporating diverse user populations, longitudinal studies, and emerging technologies such as artificial intelligence and personalized interventions. By integrating insights from interdisciplinary perspectives such as human-computer interaction, behavioral sciences, and health care informatics, the understanding of user needs and app effectiveness can be enhanced. A more standardized framework for assessing UX in mHealth apps is also a gap that needs to be addressed to facilitate comparability across studies and improve app design to maximize user engagement and health outcomes. While this analysis provides valuable insights into the fundamental features of the field, it does not explore the underlying causes or offer predictive capabilities. Future research should integrate these findings with inferential or exploratory analyses to uncover deeper insights.

## Supplementary material

10.2196/75909 Multimedia Appendix 1 Distribution of database sources and proportion of publication types in the bibliometric analysis.

10.2196/75909 Multimedia Appendix 2 Chart of the leading countries’ contributions.

10.2196/75909 Multimedia Appendix 3 Chart of the leading research entities' contributions.

10.2196/75909 Multimedia Appendix 4 Citation analysis summary showing the annual most cited publications.

10.2196/75909 Multimedia Appendix 5 The co-citation network and citation burst.

10.2196/75909 Multimedia Appendix 6 The coauthorship analysis figures and the authors' collaboration summary.

10.2196/75909 Multimedia Appendix 7 Co-words frequency analysis.

10.2196/75909 Multimedia Appendix 8 Theory-driven versus data-driven research on UXS-mHealth apps.

10.2196/75909 Multimedia Appendix 9 Keyword analysis for the 5 largest clusters.

10.2196/75909 Checklist 1 PRISMA checklist.

## Abbreviations

AHP: Analytical Hierarchy Process
HITAM: Health Information Technology Acceptance Model
MARS: Mobile App Rating Scale
MAUQ: Mobile Health App Usability Questionnaire
mHealth: mobile health
PRISMA: Preferred Reporting Items For Systematic Reviews and Meta-Analyses
PSSUQ: Post-Study System Usability Questionnaire
SLR: systematic literature review
SUS: System Usability Scale
TAM: Technology Acceptance Model
TC: total citations
TP: total publications
UTAUT: Unified Theory of Acceptance and Use of Technology
UX: user experience
UXS: User Experience And Satisfaction
