# Supplementary material for: User Experience in mHealth Research: Bibliometric Analysis of Trends and Developments (2007–2023)
Source: JMIR Mhealth Uhealth. 2025 Nov 10;13:e75909. doi: 10.2196/75909 (PMC12599265; doi:10.2196/75909)

Multimedia Appendix 1

Distribution of database sources and proportion of publication types in the bibliometric analysis.

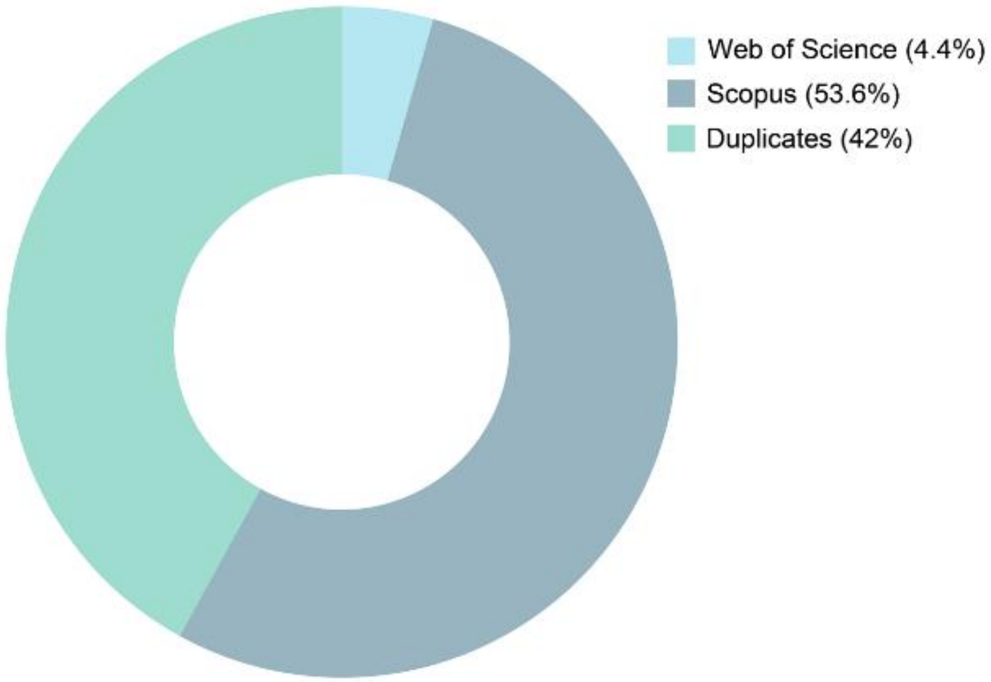

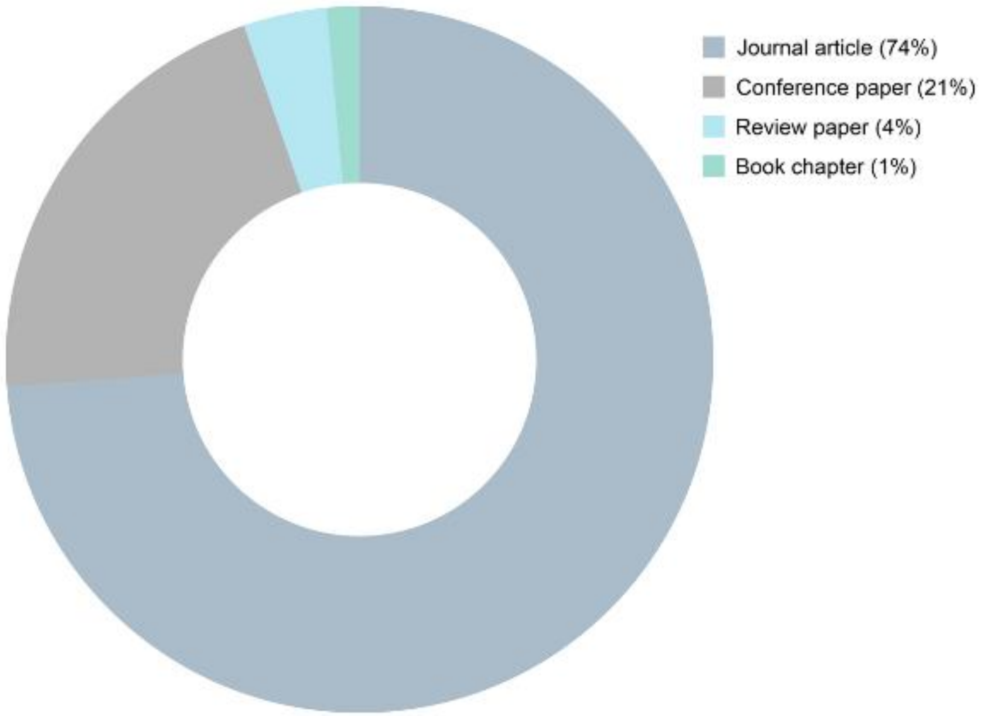

Supplement: Multimedia Appendix 1 [file mhealth-v13-e75909-s001.pdf]
