# Supplementary material for: User Experience in mHealth Research: Bibliometric Analysis of Trends and Developments (2007–2023)
Source: JMIR Mhealth Uhealth. 2025 Nov 10;13:e75909. doi: 10.2196/75909 (PMC12599265; doi:10.2196/75909)

Multimedia Appendix 2

The top 20 countries, out of 81, contributed to research on UXS-mHealthApps from 2007 to 2023. The country ranking is based on the bibliometric indicators: total publications (TP) and the number of citations received (TC).

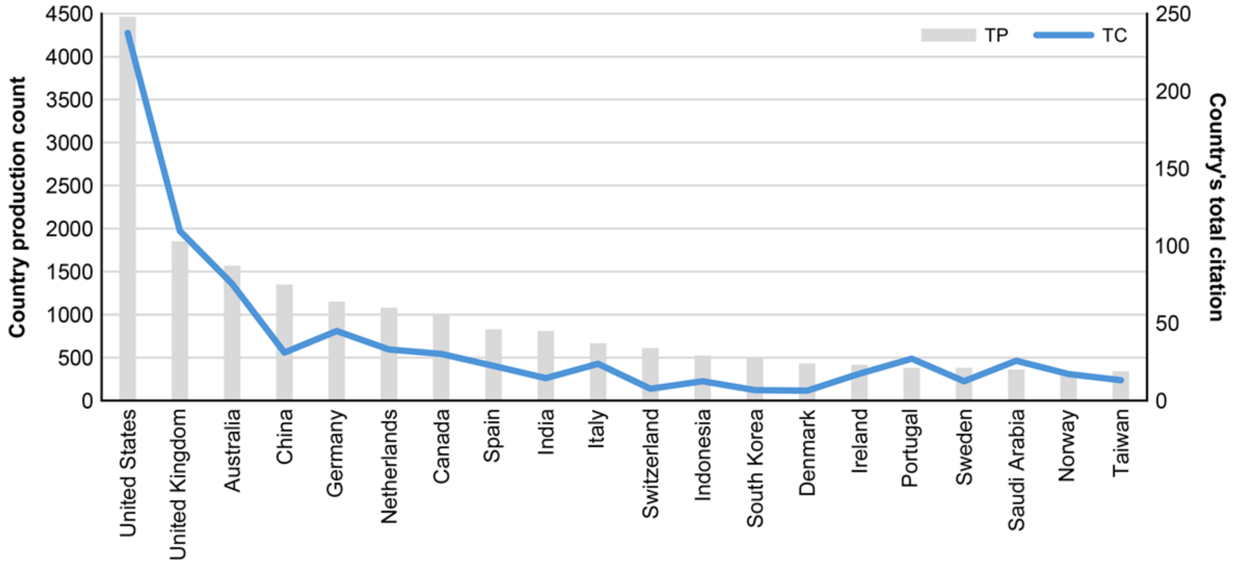

Supplement: Multimedia Appendix 2 [file mhealth-v13-e75909-s002.pdf]
