# Supplementary material for: User Experience in mHealth Research: Bibliometric Analysis of Trends and Developments (2007–2023)
Source: JMIR Mhealth Uhealth. 2025 Nov 10;13:e75909. doi: 10.2196/75909 (PMC12599265; doi:10.2196/75909)

Multimedia Appendix 3

The top 30 research entities, out of 1948, contributed to research on user experience and satisfaction with mHealth applications from 2007 to 2023. The ranking of the research entities is based on total publications and the number of citations received. The Y-axis lists the top 30 research entities that contributed to this research between 2007 and 2023, while the X-axis displays the TP (blue bars) and citations (gray bars).

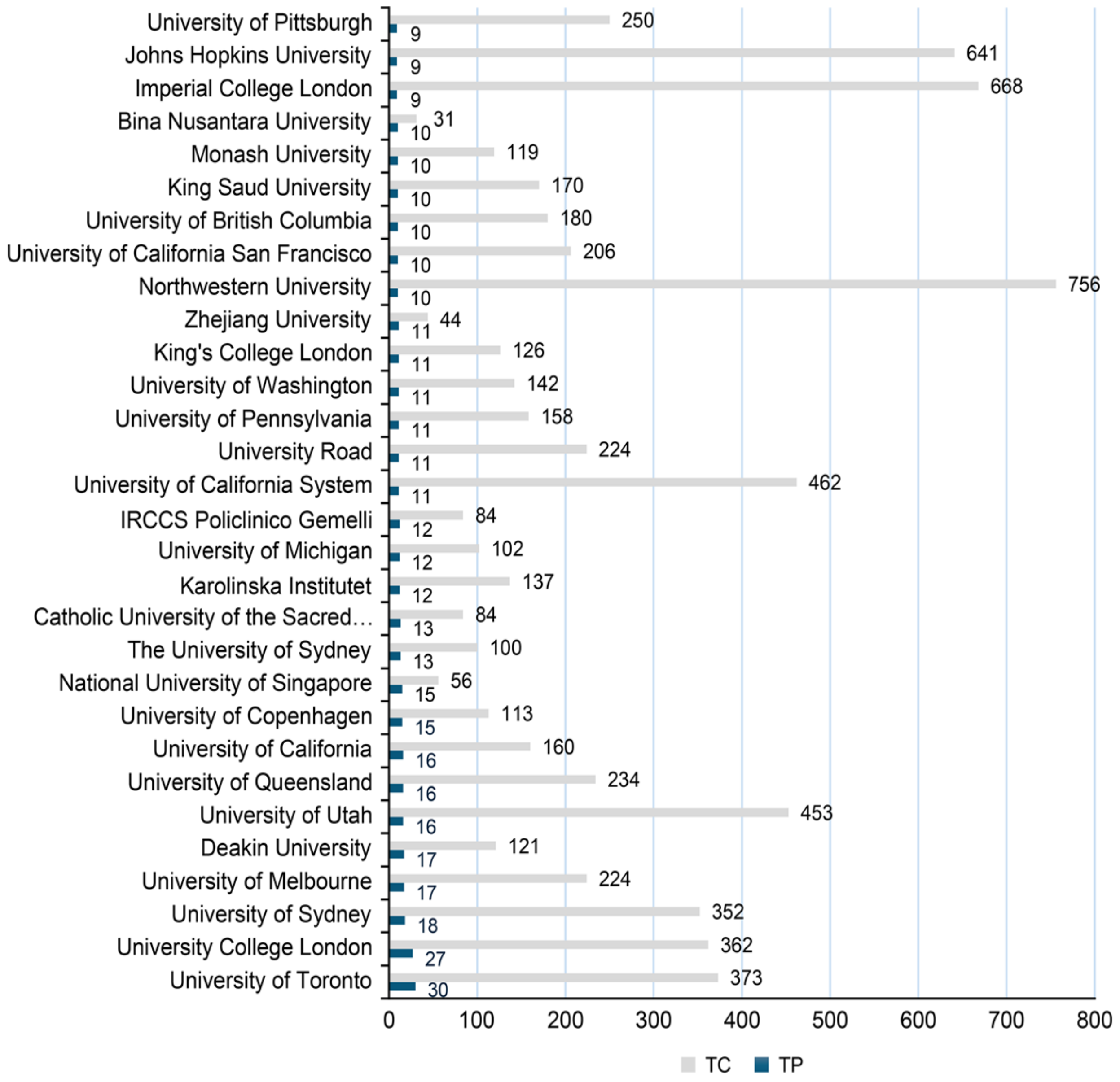

Supplement: Multimedia Appendix 3 [file mhealth-v13-e75909-s003.pdf]
