# Supplementary material for: User Experience in mHealth Research: Bibliometric Analysis of Trends and Developments (2007–2023)
Source: JMIR Mhealth Uhealth. 2025 Nov 10;13:e75909. doi: 10.2196/75909 (PMC12599265; doi:10.2196/75909)
Supplement: Multimedia Appendix 4 [file mhealth-v13-e75909-s004.pdf]

Multimedia Appendix 4

The annual most cited publications on UXS-mHealth apps from 2011 to 2023 are listed by title, author, source, country, and total citations (TC).

Year	TC	Title	Source	Country
2011	224	Status and trends of mobile health applications for iOS devices: A developer's perspective	Journal of Systems and Software	United States
2013	623	Opportunities and challenges for smartphone applications in supporting health behavior change: Qualitative study	Journal of Medical Internet Research	United Kingdom
2014	77	A Persuasive and Social mHealth Application for Physical Activity: A Usability and Feasibility Study	JMIR mHealth and uHealth	United States<br>Canada<br>Indonesia
2015	285	Keep Using My Health Apps: Discover Users' Perception of Health and Fitness Apps with the UTAUT2 Model	Telemedicine and e-Health	United States
2016	327	Mobile health apps to facilitate self-care: A qualitative study of user experiences	PLoS ONE	Australia
2017	190	A Human-centered design methodology to enhance the usability, human factors, and user experience of connected health systems: A three-phase methodology	JMIR Human Factors	Ireland<br>United States
2018	348	Using fitness trackers and smartwatches to measure physical activity in research: Analysis of consumer wrist-worn wearables	Journal of Medical Internet Research	Norway<br>Czech Republic
2019	350	Digital mental health interventions for depression, anxiety and enhancement of psychological well-being among college students: Systematic review	Journal of Medical Internet Research	United States
2020	138	Insights from user reviews to improve mental health apps	Health Informatics Journal	Canada<br>Saudi Arabia
2021	104	Short video apps as a health information source: an investigation of affordances, user experience and users' intention to continue the use of TikTok	Internet Research	China
2022	61	Blockchain-Based Trust Management Framework for Cloud Computing-Based Internet of Medical Things (IoMT): A Systematic Review	Computational Intelligence and Neuroscience	Australia
2023	36	An Overview of Chatbot-Based Mobile Mental Health Apps: Insights from App Description and User Reviews	JMIR mHealth and uHealth	United States
