# Supplementary material for: User Experience in mHealth Research: Bibliometric Analysis of Trends and Developments (2007–2023)
Source: JMIR Mhealth Uhealth. 2025 Nov 10;13:e75909. doi: 10.2196/75909 (PMC12599265; doi:10.2196/75909)
Supplement: Multimedia Appendix 5 [file mhealth-v13-e75909-s005.pdf]

Multimedia Appendix 5

The co-citation mapping for UXS-mHealthApps from 2007–2023 for the nine largest and interconnected clusters, the largest node in each cluster represents a highly influential publication, demonstrating its prominence within that area.

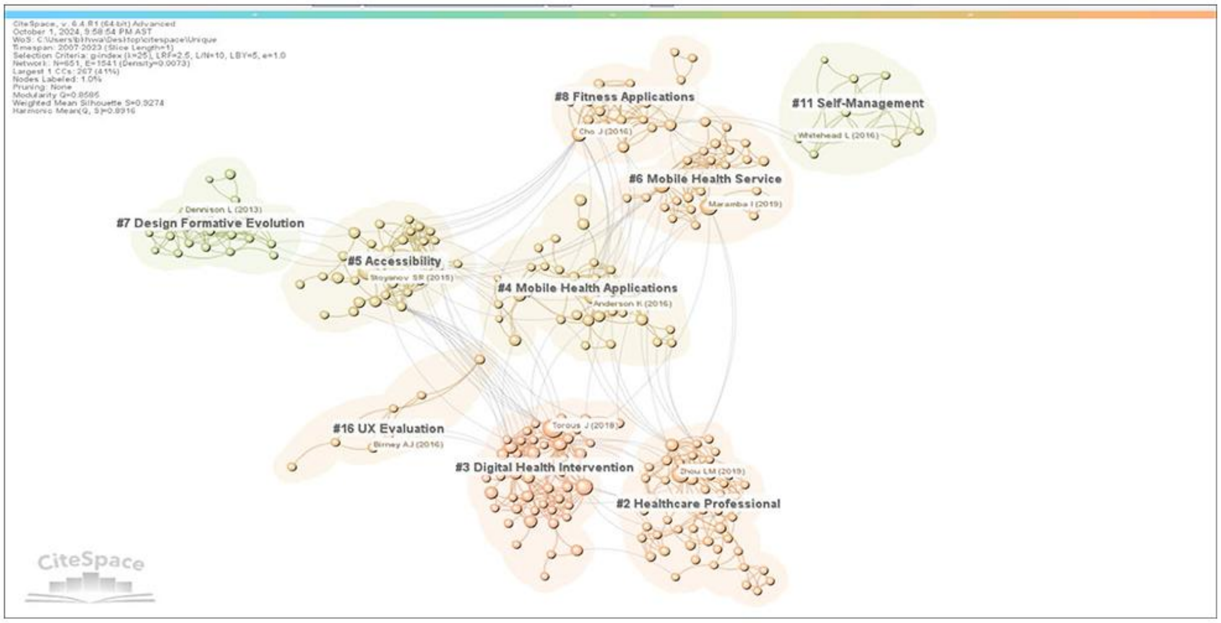

The top 8 references with the strongest citation bursts are highlighted by the analysis of co-citation analysis of the cited references on the UXS-mHealth apps between 2007–2023, showing the time window for the references that have experienced a rapid increase in citations over a specific time.

References	Year	Strength	Begin	End	2007-2023
Stoyanov SR, 2015, JMIR MHEALTH UHEALTH, V3, P0, DOI 10.2196/mhealth.3422, DOI	2015	5.6	**2017**	2020	
Anderson K, 2016, PLOS ONE, V11, P0, DOI 10.1371/journal.pone.0156164, DOI	2016	3.48	**2019**	2021	
Yardley L, 2016, AM J PREV MED, V51, P833, DOI 10.1016/j.amepre.2016.06.015, DOI	2016	3.41	**2020**	2021	
Cho J, 2016, INT J MED INFORM, V87, P75, DOI 10.1016/j.ijmedinf.2015.12.016, DOI	2016	3.41	**2020**	2021	
Torous J, 2018, EVID-BASED MENTHEAL, V21, P116, DOI 10.1136/eb-2018-102891, DOI	2018	3.16	**2020**	2023	
Zhou LM, 2019, JMIR MHEALTH UHEALTH, V7, P0, DOI 10.2196/11500, DOI	2019	3.84	**2021**	2023	
Maramba I, 2019, INT J MED INFORM, V126, P95, DOI 10.1016/j.ijmedinf.2019.03.018, DOI	2019	3.84	**2021**	2023	
Baumel A, 2019, J MED INTERNET RES, V21, P0, DOI 10.2196/14567, DOI	2019	3.84	**2021**	2023	
