# Supplementary material for: User Experience in mHealth Research: Bibliometric Analysis of Trends and Developments (2007–2023)
Source: JMIR Mhealth Uhealth. 2025 Nov 10;13:e75909. doi: 10.2196/75909 (PMC12599265; doi:10.2196/75909)
Supplement: Multimedia Appendix 6 [file mhealth-v13-e75909-s006.pdf]

Multimedia Appendix 6

The co-authorship network for the top 100 authors contributing to the UXS-mHealth app research from 2007 to 2023, revealing 10 distinct clusters for co-authorship collaboration.

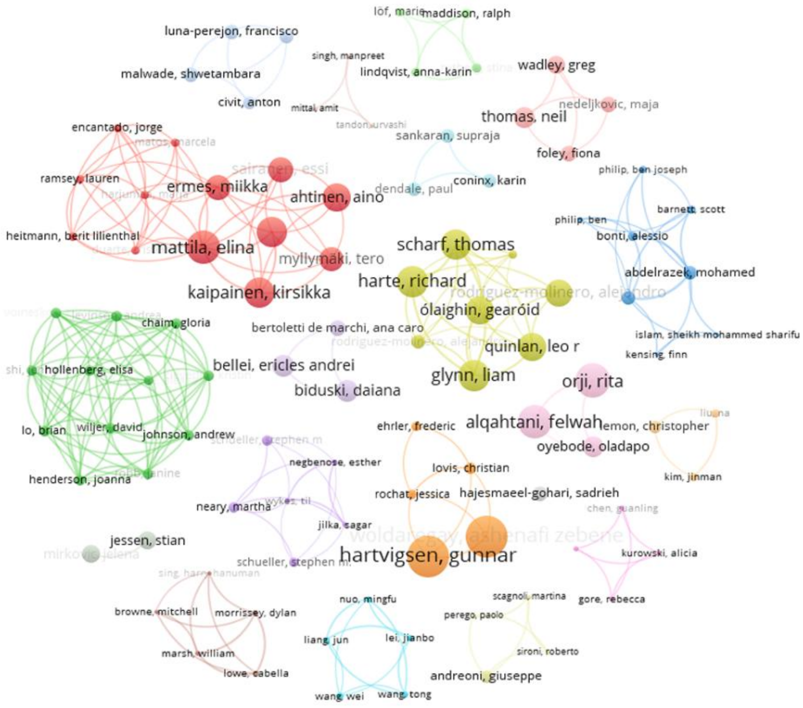

The analysis of co-authorship trends evolution over time for the top 100 authors contributing to the UXS-mHealth app research from 2007 to 2023, visualizing the emerging groups in co-authorship.

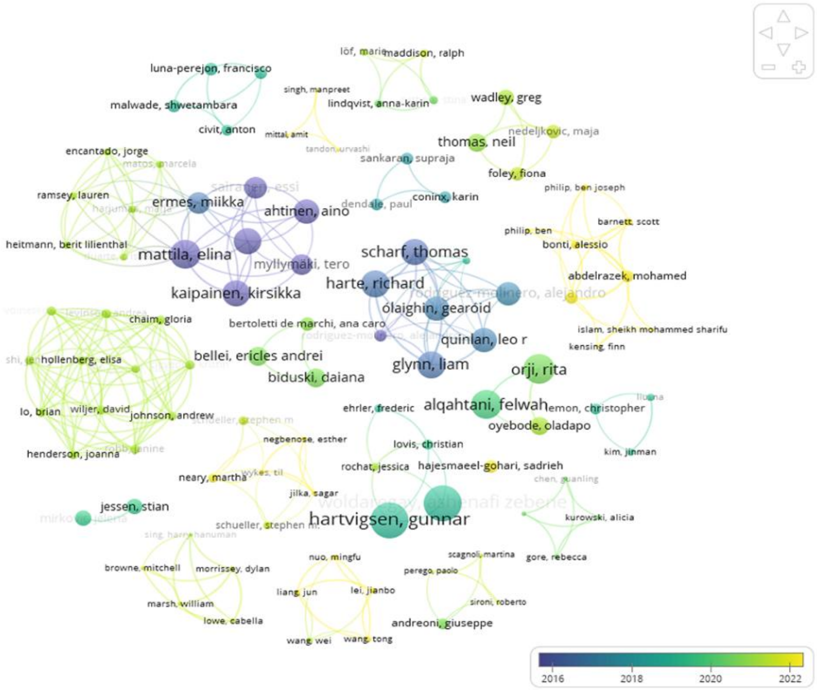

The co-citation analysis for authors reveals the most cited authors, and their strength and citation bursts over the years.

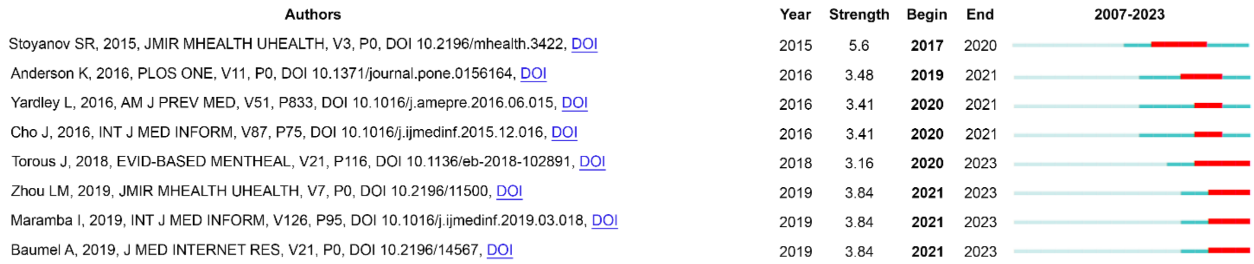

Authors	Year	Strength	Begin	End	2007-2023
Stoyanov SR, 2015, JMIR MHEALTH UHEALTH, V3, P0, DOI 10.2196/mhealth.3422, DOI	2015	5.6	**2017**	2020	
Anderson K, 2016, PLOS ONE, V11, P0, DOI 10.1371/journal.pone.0156164, DOI	2016	3.48	**2019**	2021	
Yardley L, 2016, AM J PREV MED, V51, P833, DOI 10.1016/j.amepre.2016.06.015, DOI	2016	3.41	**2020**	2021	
Cho J, 2016, INT J MED INFORM, V87, P75, DOI 10.1016/j.ijmedinf.2015.12.016, DOI	2016	3.41	**2020**	2021	
Torous J, 2018, EVID-BASED MENTHEAL, V21, P116, DOI 10.1136/eb-2018-102891, DOI	2018	3.16	**2020**	2023	
Zhou LM, 2019, JMIR MHEALTH UHEALTH, V7, P0, DOI 10.2196/11500, DOI	2019	3.84	**2021**	2023	
Maramba I, 2019, INT J MED INFORM, V126, P95, DOI 10.1016/j.ijmedinf.2019.03.018, DOI	2019	3.84	**2021**	2023	
Baumel A, 2019, J MED INTERNET RES, V21, P0, DOI 10.2196/14567, DOI	2019	3.84	**2021**	2023	

The collaboration summary of the five most impactful authors, based on citations received from authors contributing to UXS-mHealth apps research from 2007 to 2023.

Authors per Cluster	Co-authorship Themes	Countries and Research Entities
Hartvigsen, Gunnar; Woldaregay, Ashenafi Zebene; Lovis, Christian; Rochat, Jessica; Ehrler, Frederic.	mHealth for chronic diseases leverages wearable devices and self-monitoring tools to improve patient engagement and data sharing. By addressing challenges related to privacy, usability, and long-term adoption, it enhances self-management and health monitoring practices.	Arctic University of Norway, and the University Hospitals of Geneva, Switzerland.
Orji, Rita; Alqahtani, Felwah; Oyebode, Oladapo.	Persuasive technologies are being used to drive behavioral change in targeted populations. By developing digital tools and studying user engagement, these technologies aim to enhance their effectiveness for improving health outcomes.	Dalhousie University, Canada, and King Khalid University, Saudi Arabia.
Mattila, Elina; Kaipainen, Kirsikka; Lappalainen, Raimo; Ahtinen, Aino; Ermes, Miikka; Sairanen, Essi; Myllymäki, Tero; Duarte, Cristiana; Encantado, Jorge; Harjumaa, Marja; Heitmann, Berit Lilienthal; Matos, Marcela; Ramsey, Lauren.	mHealth focuses on mental well-being, stress management, and physical health by developing and evaluating wearable technologies and mobile apps. These tools assist users for managing stress, maintaining weight loss, and enhancing overall health through personalized and behavior change techniques.	Tampere University of Technology in Finland, the University of Jyväskylä in Finland, and the University of Copenhagen in Denmark.

Glynn, Liam; Harte, Richard; Scharf, Thomas; Quinlan, Leo R.; Ólaighin, Gearóid; Rodríguez-Molinero, Alejandro; Rodríguez-Molinero, Alejandro; Reixach, Elisenda.	Telemedicine and mHealth technologies emphasize remote health monitoring and digital solutions designed for elderly populations and individuals with chronic conditions. By developing sensor-based systems for tracking physical activity, detecting falls, and supporting rehabilitation, these innovations aim to improve health outcomes.	Ireland School of Medicine, and Spain University Autònoma de Barcelona.
Bellei, Ericles Andrei; Biduski, Daiana; Bertoletti de Marchi, Ana Carolina.	Human–computer interactions and usability in mHealth focus on optimizing the way users interact with digital health tools. By evaluating the usability of health applications using frameworks such as the Technology Acceptance Model (TAM), these efforts seek to enhance user experience and health outcomes.	Brock University, Canada.
