# Supplementary material for: User Experience in mHealth Research: Bibliometric Analysis of Trends and Developments (2007–2023)
Source: JMIR Mhealth Uhealth. 2025 Nov 10;13:e75909. doi: 10.2196/75909 (PMC12599265; doi:10.2196/75909)

Multimedia Appendix 7

The 30 most frequent keywords that meet the threshold of at least three occurrences, showing the rank, occurrences, links to other keywords, and the total link strength. These keywords are derived from 814 publications on UXS-mHealth apps between 2007 and 2023.

Keyword	Occurrences	Cluster	Links	Total link Strength
mobile apps	425	1	49	1035
mhealth	399	4	49	1149
user experience	218	1	46	577
mobile health	197	2	47	610
mobile phone	119	2	45	397
digital health	111	2	48	382
usability	106	5	40	351
smartphones	78	2	38	230
e-health	73	4	37	286
telemedicine	69	2	27	178
user-centered design	69	6	37	227
acceptance	68	5	37	233
mental health	63	1	34	221
self-management	60	4	33	221
qualitative research	56	3	34	180
covid-19	55	5	31	145
user satisfaction	49	5	24	110
wearable technology	41	3	29	108
engagement	38	3	28	124
adolescence	35	1	26	112
machine learning	35	1	20	68
chronic	30	4	28	122
sentiment analysis	30	1	17	57
chatbots	29	1	21	70
physical activity	28	3	23	88
gamification	26	4	23	76
artificial intelligence	24	1	16	44
behavior change	22	3	20	83
depression	21	1	20	65
intervention	21	3	22	65

The word cloud visualizes 403 out of 2,210 keywords that meet the threshold of at least three occurrences, derived from 814 publications on UXS-mHealth apps between 2007 and 2023.

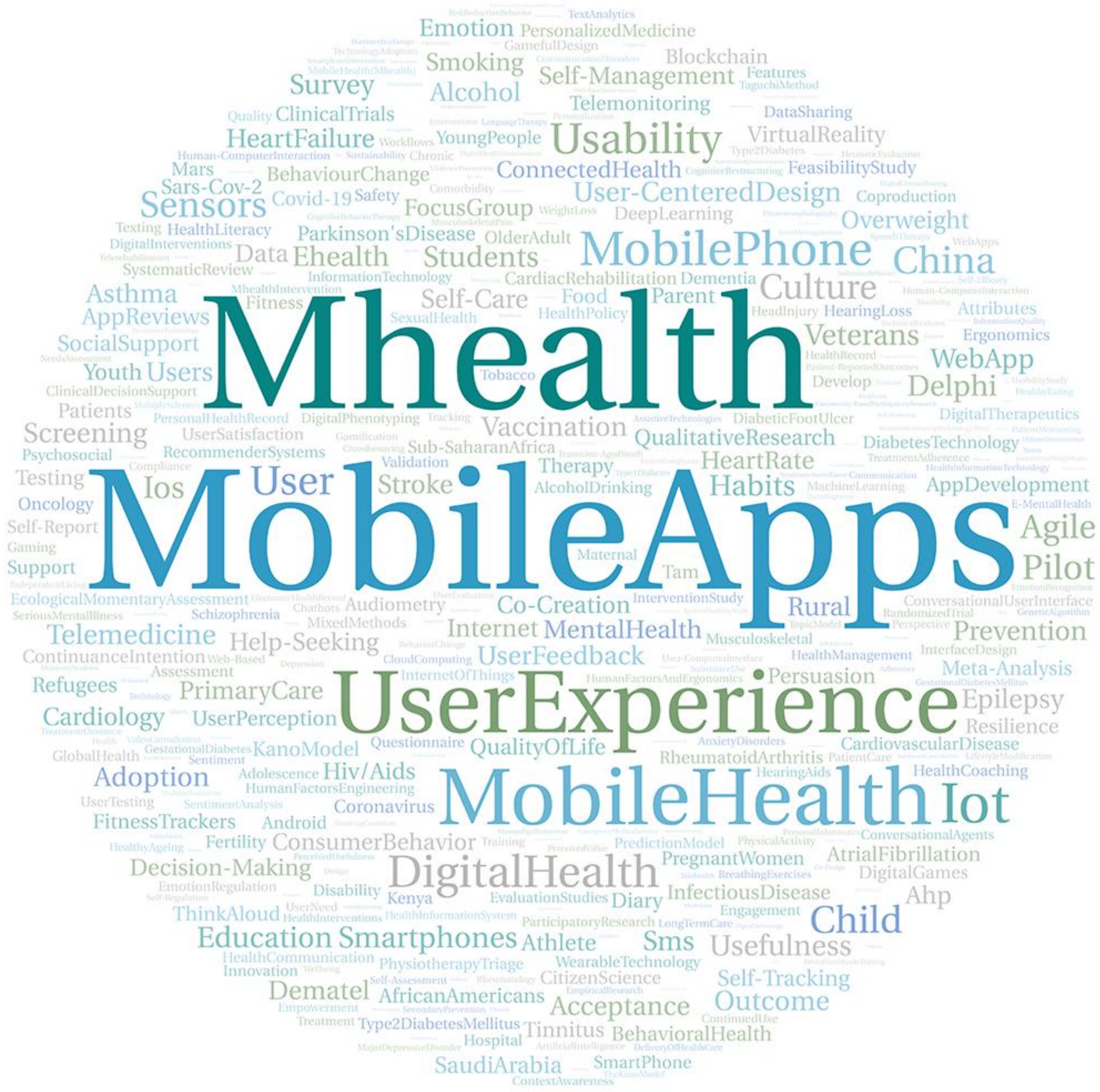

Supplement: Multimedia Appendix 7 [file mhealth-v13-e75909-s007.pdf]
