# Supplementary material for: User Experience in mHealth Research: Bibliometric Analysis of Trends and Developments (2007–2023)
Source: JMIR Mhealth Uhealth. 2025 Nov 10;13:e75909. doi: 10.2196/75909 (PMC12599265; doi:10.2196/75909)
Supplement: Multimedia Appendix 8 [file mhealth-v13-e75909-s008.pdf]

Multimedia Appendix 9

Table S 1. The most frequently used theoretical framework in research on UXS-mHealth apps from 2007 to 2023 is identified by the presence of words such as "theor*," "model," or "survey" in their title, keywords, or abstract.

Ranking	Theory	Count of studies	Percentage of studies
1.	Technology Acceptance Model (TAM)	102	38.93%
2.	Health Belief Model (HBM)	30	11.45%
3.	Unified Theory of Acceptance And Use of Technology (UTAUT)	25	9.54%
4.	Behavioral Change Theory (BCT)	23	8.78%
5.	Cognitive Behavioral Therapy (CBT)	20	7.63%
6.	Self-Determination Theory (SDT)	20	7.63%
7.	Theory of Planned Behavior (TPB)	12	4.58%
8.	Social Cognitive Theory (SCT)	10	3.82%
9.	Health Technology Assessment (HTA)	8	3.05%
10.	Gamification Theory	8	3.05%
11.	Information Systems Success Model	7	2.67%
12.	Human–Computer Interaction (HCI) Theory	6	2.29%
13.	Kano Model	6	2.29%
14.	Herzberg's 2-Factor Theory	5	1.91%
15.	Self-Management Theory	5	1.91%
16.	UX Honeycomb Model	5	1.91%
17.	Diffusion of Innovations Theory (DOI)	4	1.53%
18.	Expectation–Confirmation Model (ECM)	3	1.15%
19.	Affordance Theory	2	0.76%
20.	Co-Design Theory	2	0.76%
21.	Evolutionary Game Theory	2	0.76%
22.	Fuzzy Set Theory	2	0.76%
23.	Stimulus–Organism–Response (SOR) Theory	2	0.76%
24.	Theory of Technology Readiness And Acceptance Model	2	0.76%
25.	Crisis Management Theory	1	0.38%
26.	Individual And Family Self-Management Theory	1	0.38%
27.	Integration of Normalization Process Theory (NPT)	1	0.38%
28.	Technology Trust Theory	1	0.38%
29.	The Theory of Reasoned Action	1	0.38%
The Studies include theoretical keywords in the title, keywords, and abstract		262	32.23%

Table S 2. A summary of the use of terms such as “machine learning,” “sentiment analysis,” and “artificial intelligence” in the titles, keywords, or abstracts of studies.

**Term**	**Count of studies**	**Percentage of studies**	**Initial publication year**	**Most cited studies**
Machine learning	34	4.18%	2017	[13, 100]
Sentiment analysis	42	5.17%	2018	[101, 102]
Artificial intelligence	25	3.08%	2019	[103, 104]
