# Supplementary material for: User Experience in mHealth Research: Bibliometric Analysis of Trends and Developments (2007–2023)
Source: JMIR Mhealth Uhealth. 2025 Nov 10;13:e75909. doi: 10.2196/75909 (PMC12599265; doi:10.2196/75909)
Supplement: Multimedia Appendix 9 [file mhealth-v13-e75909-s009.pdf]

Multimedia Appendix 8

Cluster 1 comprises 259 keywords in total, with the top 50 listed in Table 1. The first network in Figure 1 presents the co-occurrence analysis of all keywords in Cluster 1, while Figure 2 focuses on those that meet the threshold of a minimum of three co-occurrences.

Table 1. List of the top 50 most frequent keywords in cluster1.

Keyword	Occurrences	Total link strength	Keyword	Occurrences	Total link strength
1. mhealth	37	190	26. evaluation studies	2	11
2. mobile apps	22	125	27. fitness trackers	2	10
3. mobile phones	12	66	28. gamification	2	13
4. user experience	9	44	29. human computer interaction	2	10
5. qualitative	8	53	30. low engagement	2	8
6. e-health	7	45	31. machine learning	2	7
7. focus groups	5	30	32. medication adherence	2	8
8. self-management	5	36	33. mental health apps	2	8
9. digital health	4	24	34. mindfulness	2	8
10. mental health	4	17	35. motivation	2	10
11. telemedicine	4	23	36. pediatrics	2	10
12. user satisfaction	4	23	37. physical activity	2	7
13. chronic disease	3	24	38. psychosis	2	8
14. exercise	3	20	39. quality of life	2	10
15. health and fitness apps	3	12	40. review	2	6
16. health behavior	3	16	41. sentiment analysis	2	8
17. obesity	3	21	42. sms	2	9
18. well-being	3	22	43. stress	2	15
19. artificial intelligence	2	9	44. technology	2	6
20. breast cancer	2	10	45. technology acceptance	2	14
21. chatbot	2	9	46. text analytics	2	8
22. co-design	2	18	47. text messaging	2	9
23. cognitive behavioral therapy	2	13	48. usability	2	13
24. consumer behavior	2	8	49. user perception	2	10
25. digital intervention	2	8	50. weight loss	2	6

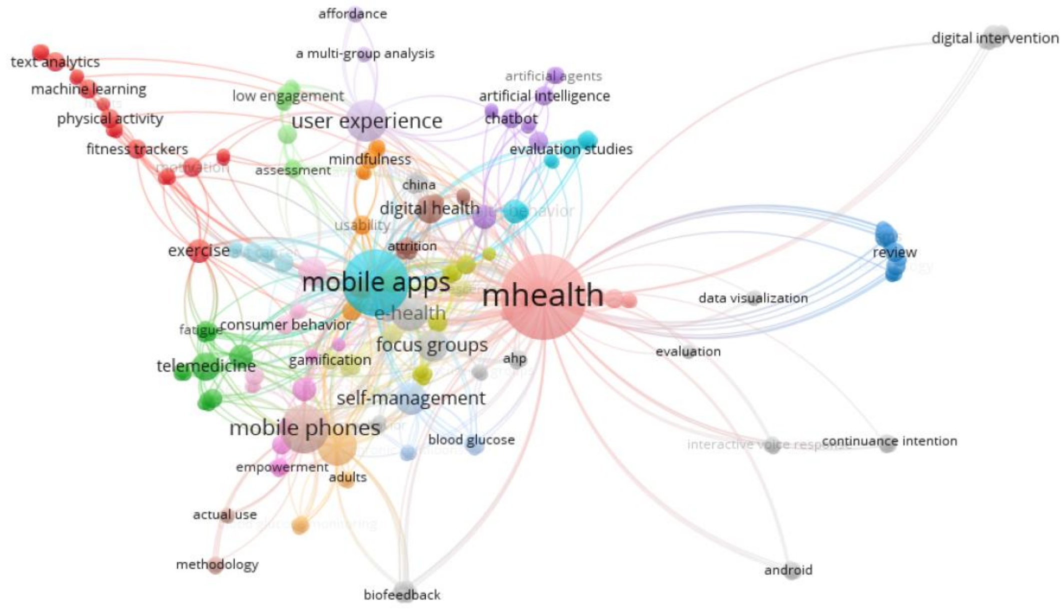

Figure 1. The keywords co-occurrence analysis of all keywords in Cluster 1.

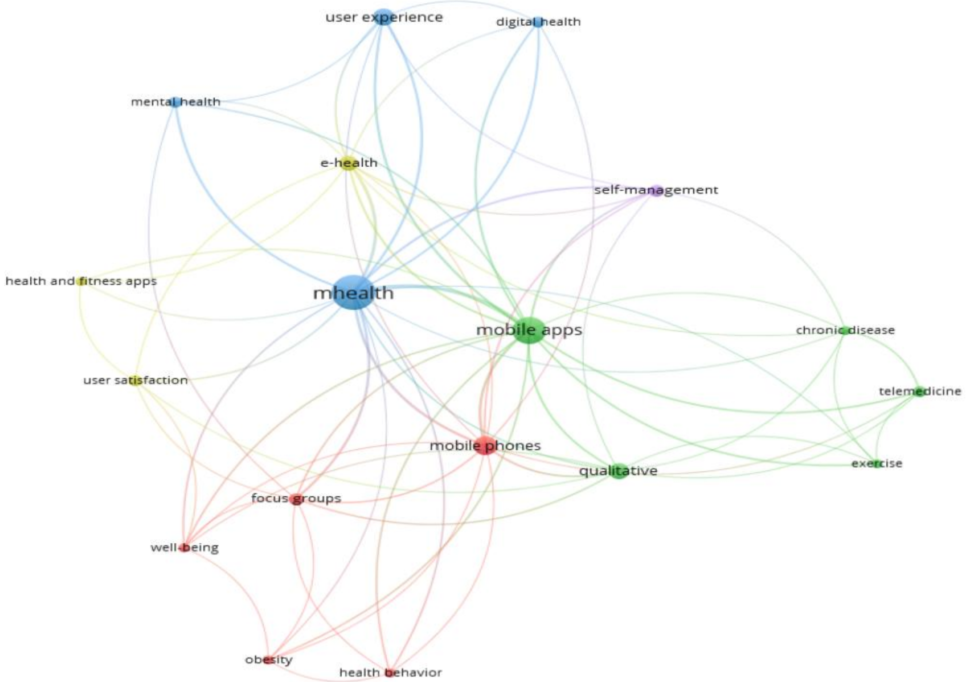

Figure 2. The co-occurrence analysis of Cluster 1 includes 18 keywords that meet the minimum threshold of three occurrences.

Cluster 2 comprises a total of 282 keywords, with the top 50 listed in Table 2. The first network in Figure 3 presents the co-occurrence analysis of all keywords in Cluster 2, while Figure 4 focuses on those that meet the threshold of at least three co-occurrences.

Table 2. List of the top 50 most frequent keywords in cluster2.

Keyword	Occurrences	Total link strength	Keyword	Occurrences	Total link strength
1. mhealth	48	282	26. chronic pain	2	13
2. mobile apps	23	135	27. co-design	2	18
3. user experience	15	94	28. covid-19	2	17
4. ehealth	11	77	29. depression	2	10
5. self-management	11	69	30. engagement	2	10
6. telemedicine	8	51	31. evaluation	2	7
7. mobile phones	7	44	32. focus group	2	17
8. mental health	6	28	33. focus groups	2	12
9. usability	6	38	34. health behavior	2	13
10. gamification	5	28	35. mars	2	17
11. chatbot	4	28	36. medication adherence	2	9
12. digital health	4	23	37. mental health apps	2	10
13. cancer	3	14	38. mobile phone app	2	18
14. cognitive behavioral therapy	3	17	39. patients	2	17
15. health promotion	3	24	40. pediatrics	2	11
16. obesity	3	17	41. qualitative research	2	11
17. physical activity	3	12	42. quality of life	2	10
18. technology	3	23	43. smartphone apps	2	11
19. telehealth	3	26	44. technology acceptance	2	16
20. usability testing	3	19	45. type 2 diabetes mellitus	2	12
21. adolescent	2	9	46. user satisfaction	2	12
22. adolescents	2	15	47. wearables	2	12
23. alcohol	2	11	48. well-being	2	14
24. children	2	13	49. academic libraries	1	5
25. chronic disease	2	15	50. acceptability	1	8

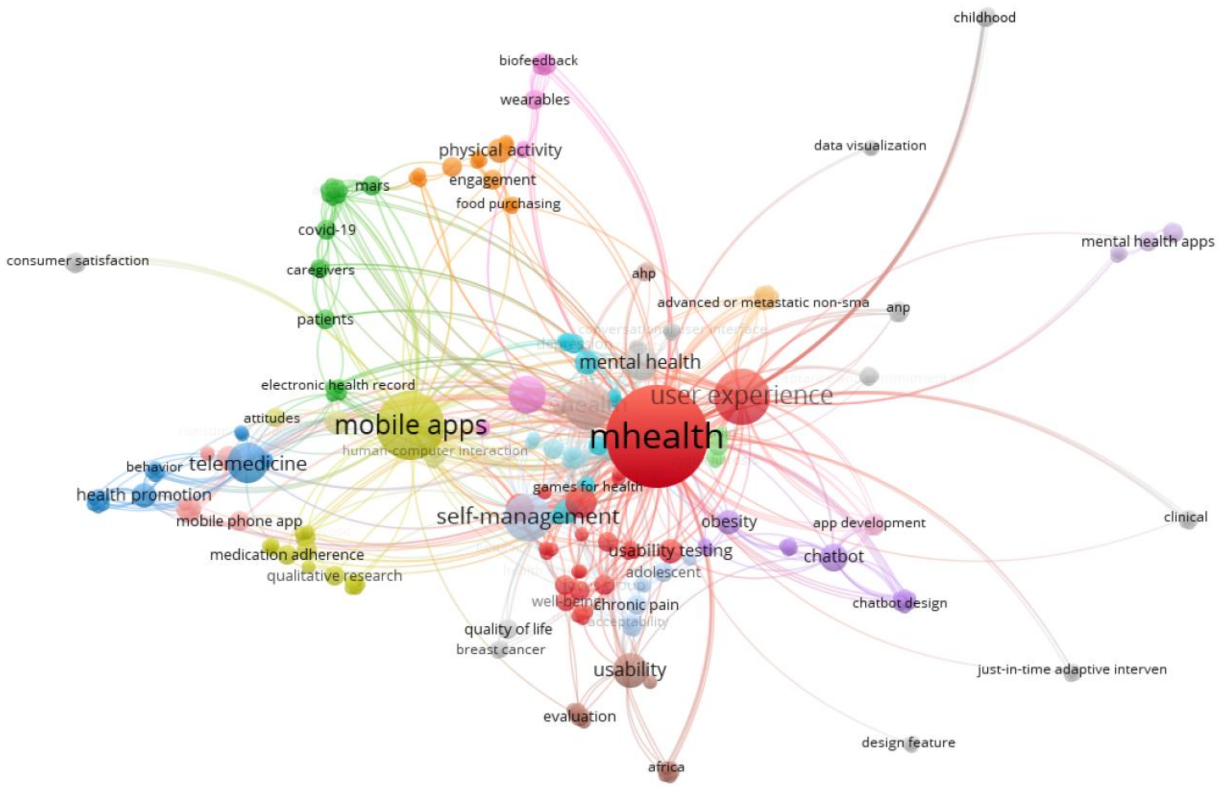

Figure 3. The keywords co-occurrence analysis of all keywords in Cluster 2.

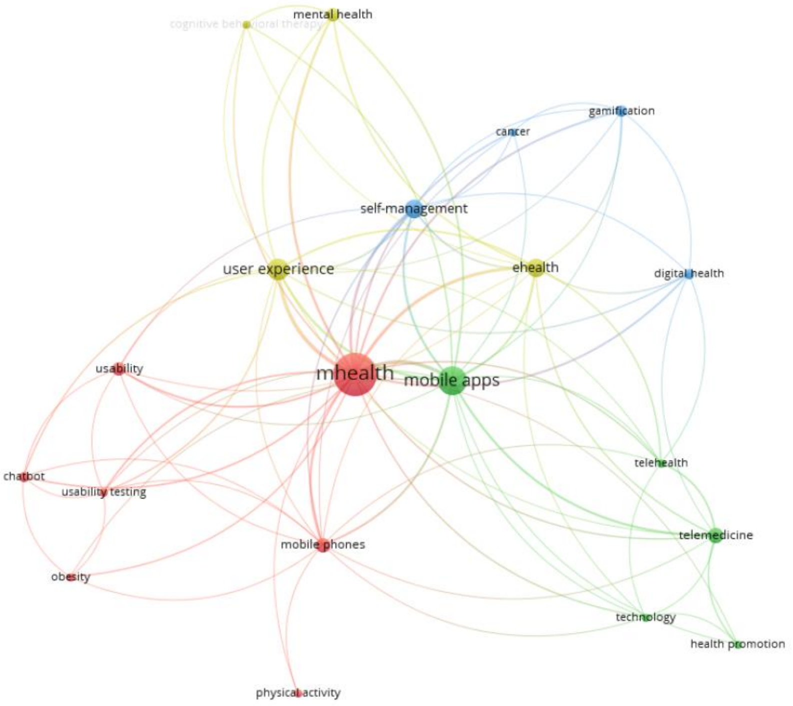

Figure 4. The co-occurrence analysis of Cluster 2 includes 20 keywords that meet the minimum threshold of three occurrences.

Cluster 3 comprises 300 keywords in total, with the top 50 listed in Table 3. The first network in Figure 1 presents the co-occurrence analysis of all keywords in Cluster 3, while Figure 2 focuses on those that meet the threshold of a minimum of three co-occurrences.

Table 3. List of the top 50 most frequent keywords in cluster 3.

Keyword	Occurrences	Total link strength	Keyword	Occurrences	Total link strength
1. mhealth	50	329	26. acceptance and commitment therapy	2	13
2. mobile apps	24	174	27. adherence	2	16
3. mobile phone	15	105	28. aged	2	18
4. mobile phones	10	72	29. anxiety	2	14
5. user experience	10	67	30. behavior change	2	14
6. ehealth	9	75	31. blood pressure	2	15
7. telemedicine	9	76	32. children	2	11
8. usability	9	76	33. cognitive behavioral therapy	2	11
9. digital health	8	61	34. connected health	2	17
10. covid-19	6	46	35. contact tracing	2	16
11. mental health	6	40	36. depression	2	14
12. self-management	6	33	37. diabetes	2	11
13. usability testing	5	29	38. elderly	2	20
14. adolescents	4	26	39. electronic health record	2	16
15. engagement	4	38	40. feasibility	2	15
16. telehealth	4	30	41. health apps	2	15
17. user-centered design	4	30	42. health care	2	14
18. chatbot	3	21	43. health education	2	15
19. heart failure	3	15	44. health promotion	2	17
20. hypertension	3	11	45. human factors	2	17
21. pediatrics	3	22	46. internet	2	15
22. physical activity	3	27	47. internet intervention	2	9
23. public health	3	21	48. lifestyle	2	15
24. stress	3	24	49. meta-analysis	2	15
25. technology	3	25	50. monitoring	2	19

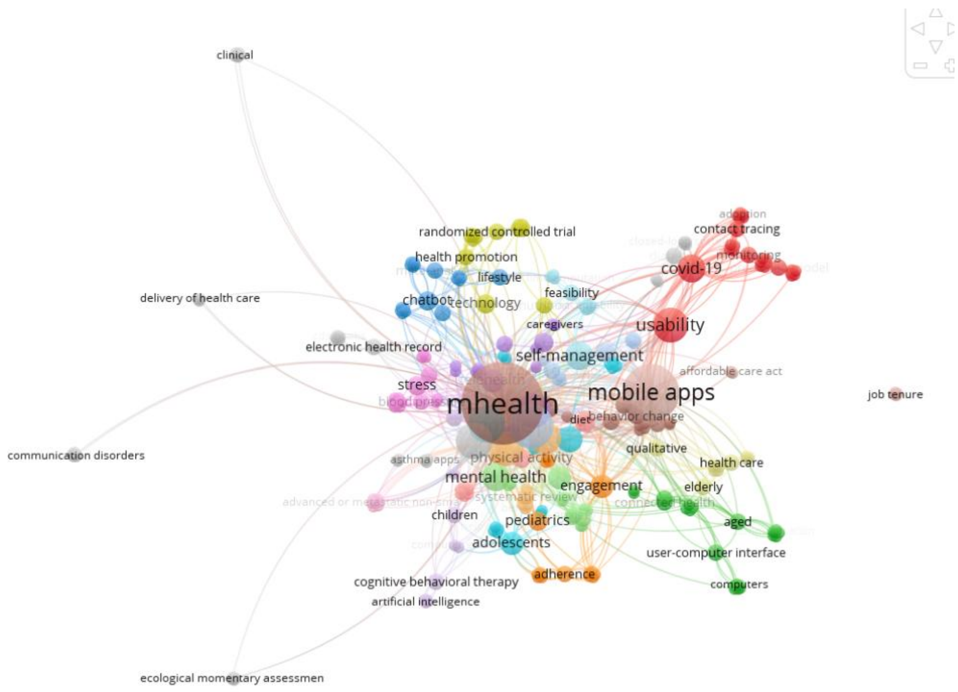

Figure 5. The keywords co-occurrence analysis of all keywords in Cluster 3.

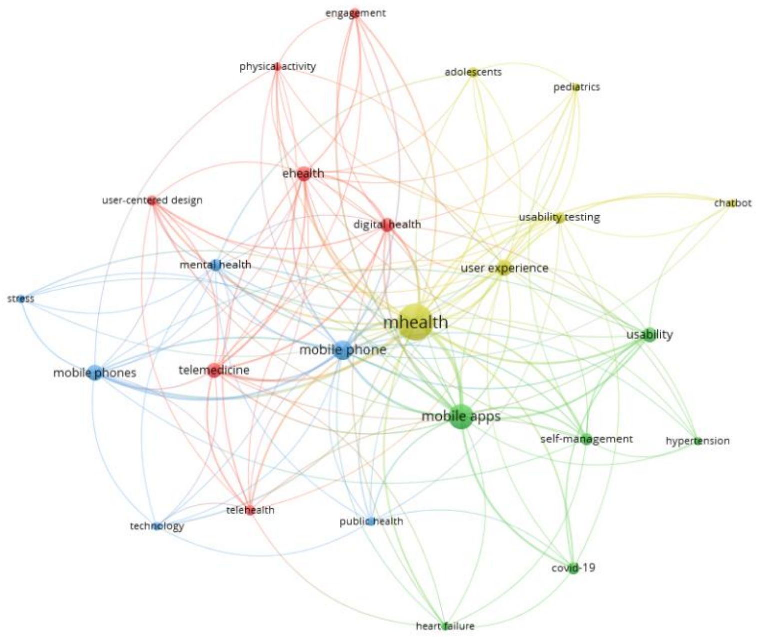

Figure 6. The co-occurrence analysis of Cluster 3 includes 25 keywords that meet the minimum threshold of three occurrences.

Cluster 4 comprises 288 keywords in total, with the top 50 listed in Table 4. The first network in Figure 7 presents the co-occurrence analysis of all keywords in Cluster 4, while Figure 8 focuses on those that meet the threshold of a minimum of three co-occurrences.

Table 4. List of the top 50 most frequent keywords in cluster 4.

Keyword	Occurrences	Total link strength	Keyword	Occurrences	Total link strength
1. mhealth	24	167	26. older adults	2	11
2. mobile apps	16	100	27. satisfaction	2	7
3. mobile health	16	134	28. self-management	2	24
4. mobile phone	10	83	29. stress	2	14
5. user experience	9	54	30. telemedicine	2	14
6. digital health	6	55	31. text analytics	2	8
7. user satisfaction	6	29	32. user-centered design	2	8
8. wearable	6	55	33. acceptability	1	16
9. mbile phone	5	43	34. actor-to-actor interaction	1	6
10. usability	5	39	35. adherence	1	16
11. acceptance and commitment therapy	3	19	36. aged	1	5
12. machine learning	3	15	37. agoraphobia	1	5
13. physical activity	3	23	38. agricultural safety and health	1	5
14. technology acceptance	3	19	39. alcohol drinking	1	8
15. augmented reality	2	11	40. ambient intelligence	1	5
16. continuance intention	2	10	41. anesthesiology	1	5
17. continued use	2	16	42. anterior cruciate ligament reconstruction	1	4
18. decision-making	2	22	43. antipsychotic	1	3
19. e-health	2	10	44. attitude	1	16
20. empirical research	2	10	45. attributes	1	3
21. exercise	2	12	46. attrition	1	10
22. feasibility study	2	10	47. background music service	1	5
23. fitness applications	2	16	48. behavior	1	5
24. internet of things	2	12	49. blood pressure	1	17
25. mental health	2	22	50. body temperature	1	17

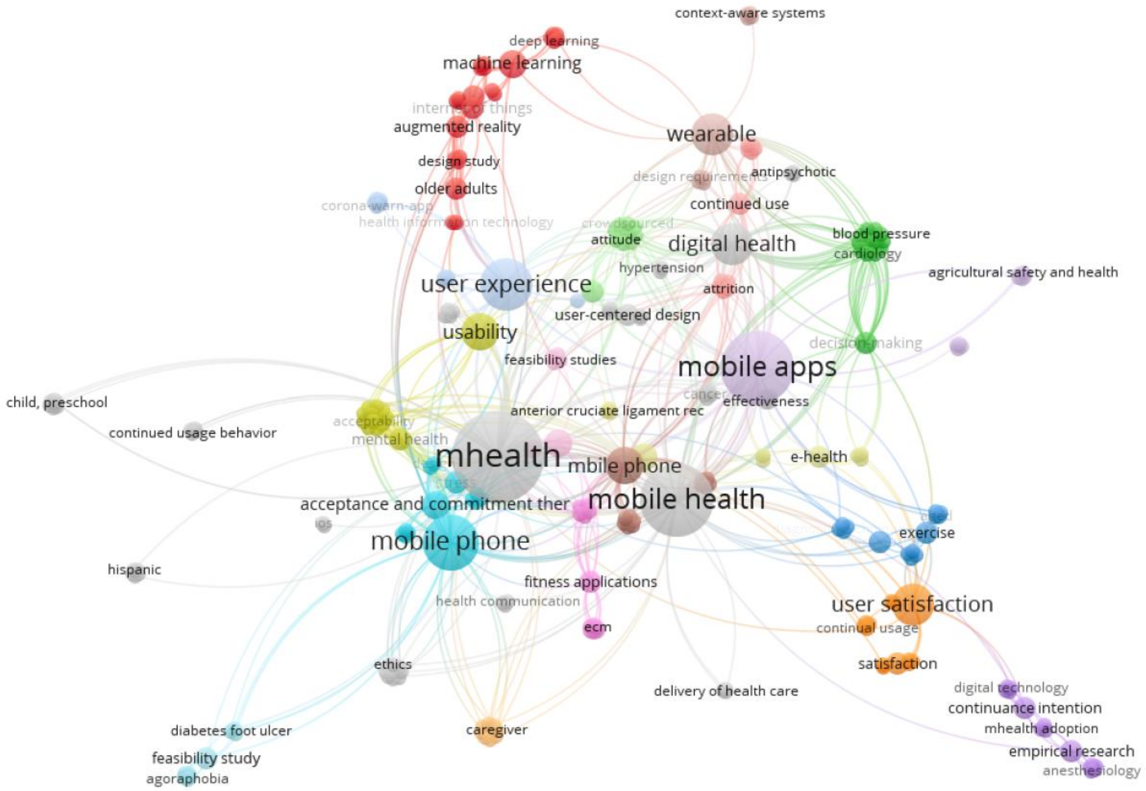

Figure 7. The keywords co-occurrence analysis of all keywords in Cluster 4.

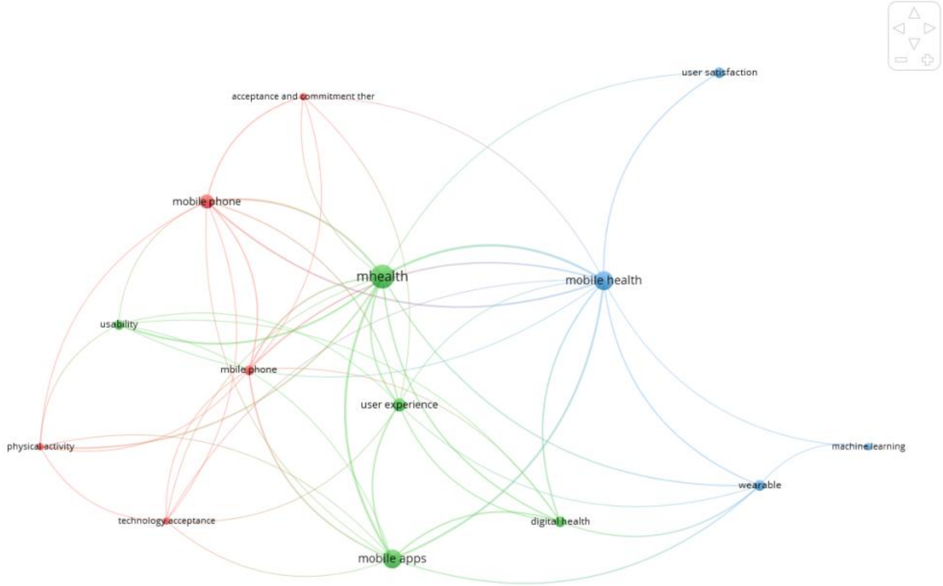

Figure 8. The co-occurrence analysis of Cluster 4 includes 14 keywords that meet the minimum threshold of three occurrences.

Cluster 5 comprises 164 keywords in total, with the top 50 listed in Table 5. The first network in Figure 9 presents the co-occurrence analysis of all keywords in Cluster 5, while Figure 10 focuses on those that meet the threshold of a minimum of three co-occurrences.

Table 5. List of the top 50 most frequent keywords in cluster 5.

Keyword	Occurrences	Total link strength	Keyword	Occurrences	Total link strength
1. mobile apps	10	51	26. chatbots	1	7
2. mhealth	9	43	27. children	1	4
3. user experience	8	43	28. chronic diseases	1	4
4. usability	6	34	29. chronic kidney disease	1	4
5. mobile phones	5	19	30. clinical decision support	1	7
6. telemedicine	4	15	31. clinical decision support system	1	3
7. user-centered design	4	16	32. co-design	1	3
8. digital health	3	16	33. communication	1	5
9. behavior change	2	12	34. communication channel	1	4
10. human factors	2	16	35. communities-led initiatives	1	5
11. human factors and ergonomics	2	8	36. community health workers	1	4
12. taguchi method	2	8	37. community-based participatory research	1	9
13. accidental falls	1	9	38. community-led initiatives	1	4
14. acufenometry	1	4	39. computerised decision support system	1	5
15. adherence	1	5	40. confidentiality	1	4
16. aged	1	9	41. consent	1	4
17. assistive technologies	1	7	42. coronavirus disease 2019	1	4
18. assistive technology	1	9	43. criteria-based dispatch	1	5
19. audiometry	1	4	44. data security	1	4
20. benchmarking	1	4	45. decision aids	1	7
21. big data	1	5	46. depression	1	4
22. business intelligence	1	3	47. design	1	9
23. cardiovascular disease	1	3	48. design and evaluation	1	5
24. cardiovascular disease risk assessment	1	4	49. device	1	4
25. cardiovascular risk management	1	5	50. diabetes self-management	1	4

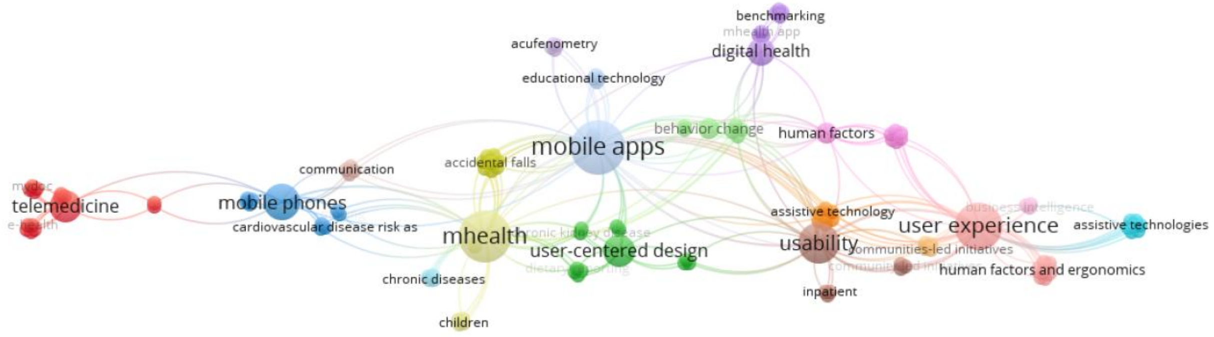

Figure 9. The keywords co-occurrence analysis of all keywords in Cluster 5.

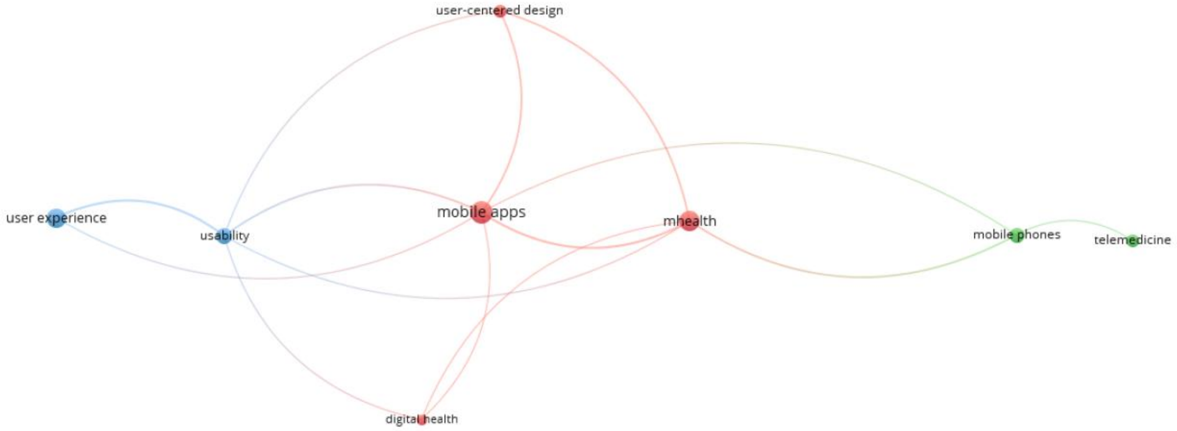

Figure 10. The co-occurrence analysis of Cluster 5 includes 8 keywords that meet the minimum threshold of three occurrences.
